# Artificial intelligence-based biomarkers for the diagnosis and treatment of neurological conditions: a narrative review

**DOI:** 10.1186/s13041-026-01287-1

**Published:** 2026-03-07

**Authors:** Adam Ben-Jaafar, Abhishek Sinha, Princess Afia Nkrumah-Boateng, Subham Roy, Vivek Sanker, Siham Mohamed, Francis Sarfo-Adu, Syed Hasham Ali, Andrew Awuah Wireko

**Affiliations:** 1 School of Medicine, University College Dublin, Belfield, Dublin 4, Ireland; 2 University of Glasgow Medical School, Glasgow, UK; 3 University of Ghana Medical School, Accra, Ghana; 4 Hull York Medical School, University of York, York, UK; 5 Department of Neurosurgery, Stanford University, Stanford, CA USA; 6 Independent Researcher, London, UK; 7 Planaria Medical Agency, London, England, UK; 8 Faculty of Medicine, Dow Medical College, Dow University of Health Sciences, Karachi, Pakistan; 9 Department of Research, Toufik World Organization, Sumy, Ukraine

**Keywords:** Artificial intelligence, AI-based biomarker, Biomarker discovery, Drug discovery, Neurological diseases, Molecular neuroscience, Neuro-informatics

## Abstract

Artificial intelligence (AI) is transforming biomarker discovery in neurology by overcoming key limitations of conventional approaches that are often slow, reductionist, and unable to integrate complex multimodal data. In this narrative review, we searched the data bases; PubMed, Scopus, IEEE Xplore, CINAHL, Embase and the Cochrane Library from inception to 2025 to evaluate how AI supports biomarker identification, diagnosis, prognostication, and treatment stratification across neurovascular, neurodegenerative, neuro-oncological and seizure disorders. Evidence demonstrates that AI-driven imaging and multi-omics biomarkers can detect disease earlier, improve prediction accuracy, and support personalised care. For example, AI models improve stroke outcome prediction beyond conventional scores, identify intracranial aneurysms with sensitivities exceeding 90%, predict conversion from mild cognitive impairment to Alzheimer’s disease with accuracies approaching 85–90%, and extract radiogenomic biomarkers in gliomas that outperform traditional diagnostic strategies. However, real-world translation remains constrained by dataset bias, limited external validation, interpretability challenges, and gaps in generalisability, particularly in underrepresented populations. Overall, AI-driven biomarker discovery offers a powerful pathway toward precision neurology, with the greatest impact expected when technical innovation is paired with robust clinical validation, regulatory integration, and equitable data representation.

**Supplementary Information:**

The online version contains supplementary material available at 10.1186/s13041-026-01287-1.

## Introduction

Neurological disorders are responsible for approximately 3.4 billion cases of ill health globally and account for over 443 million disability-adjusted life years, reflecting an 18.2 percent increase since 1990 [1]. This burden is disproportionately concentrated in low-resource settings but is also rising in high-income countries due to population ageing and modifiable risk factors, including overweight and obesity [2, 3]. Despite this scale, current diagnostic and therapeutic approaches remain limited by biological complexity, delayed recognition, restricted biomarker availability, and barriers such as the blood–brain and blood–spinal cord barriers, which contribute to misdiagnosis and suboptimal treatment response [4–6]. These challenges underscore the urgent need for earlier, more sensitive, and biologically informed biomarkers capable of improving disease stratification and guiding precision-based interventions across neurological conditions [5, 7].

Alongside these developments, artificial intelligence (AI) is becoming an increasingly important influence in neurological research and clinical care. Using machine learning (ML) algorithms and neural networks, it can handle large and complex datasets more effectively than traditional methods. This capability offers opportunities to improve diagnostic accuracy and to support clinical decision-making [8]. There is also growing evidence that AI may help predict survival and other patient outcomes, though these predictions remain highly dependent on the quality of the data and the clinical context in which they are applied. In addition to these applications, AI is being investigated for its potential to assist in real-time diagnosis and to guide treatment strategies that are more adaptive and tailored to the individual patient. In neuro-oncology, and particularly in the study and management of gliomas, AI has shown considerable promise. Recent work suggests that it can improve the interpretation of magnetic resonance imaging (MRI) by detecting subtle abnormalities and extracting clinically relevant features that might not be visible to the human eye. Such approaches have the potential to improve tumour grading, prognostic assessment, and the monitoring of treatment response [9]. Techniques including ML, deep learning (DL), and computer vision are now being applied not only for imaging interpretation but also for more detailed tumour characterisation, predictive modelling, and treatment planning.

ML, a subset of AI, utilises statistical models to recognise patterns in imaging and genomic data. DL, a subset of ML, further utilises multilayered artificial neural networks (ANNs) to extract complex features from unstructured data, while computer vision transforms visual data into analysable medical images. Together, these aid the advancement of care for neurological disorders [9, 10]. Building on these developments, researchers are increasingly applying AI to support biomarker discovery. Much of the early work in this area has been carried out in oncology, where AI has been shown to accelerate the identification, validation, and clinical translation of biomarkers [11]. Researchers are beginning to apply these approaches in neurology, where AI is used to bring together data from imaging, genomics, proteomics, and clinical records to support the discovery of new biomarkers for neurological disorders [12]. Taken together, these findings highlight the potential of AI to address many of the barriers that have slowed progress in traditional biomarker research.

Although existing narrative and systematic reviews have explored either AI techniques or individual biomarker modalities, most remain narrowly focused on single disease groups, isolated imaging modalities, or specific data domains. These reviews tend to evaluate AI performance in diagnostic classification without fully addressing its role across the entire biomarker pipeline; including discovery, prognostication, treatment stratification and drug development. Few reviews integrate neurovascular, neurodegenerative, neuro-oncological and seizure disorders within a unified framework, nor do they synthesise evidence across multimodal datasets such as neuroimaging, multi-omics, electrophysiology and clinical records. This leaves an important gap in understanding how AI-driven biomarkers can support precision medicine in neurology at scale.

This narrative review therefore aims to evaluate the role of AI in biomarker discovery for neurological disorders, particularly in terms of its potential to improve diagnostic accuracy, prognostication and therapeutic strategies, thus advancing precision medicine in neurology.

## Methodology

This narrative review was conducted using the Scale for the Assessment of Narrative Reviews (SANRA) [13], with the aim of evaluating the use of AI in biomarker discovery and prognosis for neurological disorders. The detailed search string provided in the appendix enabled a comprehensive and targeted review of the literature to be conducted.

### Eligibility criteria

To be eligible for inclusion, any piece of research must satisfy the following conditions: 1) focus on or mention AI-driven biomarkers and 2) describe the diagnostic or therapeutic utility of these biomarkers in patients with CNS disorders. These relatively broad criteria allowed us to achieve our stated goal of integrating neuro-oncological, neurodegenerative, neurodevelopmental, neurovascular and seizure disorders into a unified framework that synthesises evidence from multiple datasets. To this end, the inclusion criteria permitted various study designs and the inclusion of both quantitative and qualitative data. This included observational studies, case–control studies, cohort studies, randomised controlled trials, systematic reviews and meta-analyses. Conference abstracts without full papers, non-peer-reviewed studies, blog posts, preprints, and papers in languages other than English were excluded from the review. To maximise data inclusion, no strict sample size limit was imposed. Additionally, no prioritisation rubric was utilised and, while the authors tried to be exhaustive in their exploration of neurological conditions, they were ultimately restricted by the paucity of literature on rare conditions in the scientific literature. The authors anticipated that there would be instances where literature on AI-based biomarkers was not present for certain rare conditions, which would ultimately lead to exclusions from the review.

### Search strategy

Relevant literature from inception to 2025 was searched for using PubMed/Medline, Scopus, Embase, CINAHL Plus, IEEE Xplorer and the Cochrane Library. The search terms used included, but were not limited to, 'artificial intelligence', 'machine learning', 'deep learning', 'AI-based biomarkers', 'multi-omics integration', 'neuroimaging biomarkers', 'biomarker discovery', 'neurological disorders', 'CNS disorders', 'neurovascular diseases', 'neurodegenerative diseases', 'neuro-oncological diseases', and 'neurodevelopmental disorders'. Several iterations of these terms and of specific neurological diseases and disorders within broad categories were also employed as search terms. Search string modifiers such as Medical Subject Headings and asterisks were used according to the database in order to maximise the scope of the search results. The complete search strings used for each database are tabulated in supplementary Table 1.

### Article selection and assessment

**Fig. 1:**
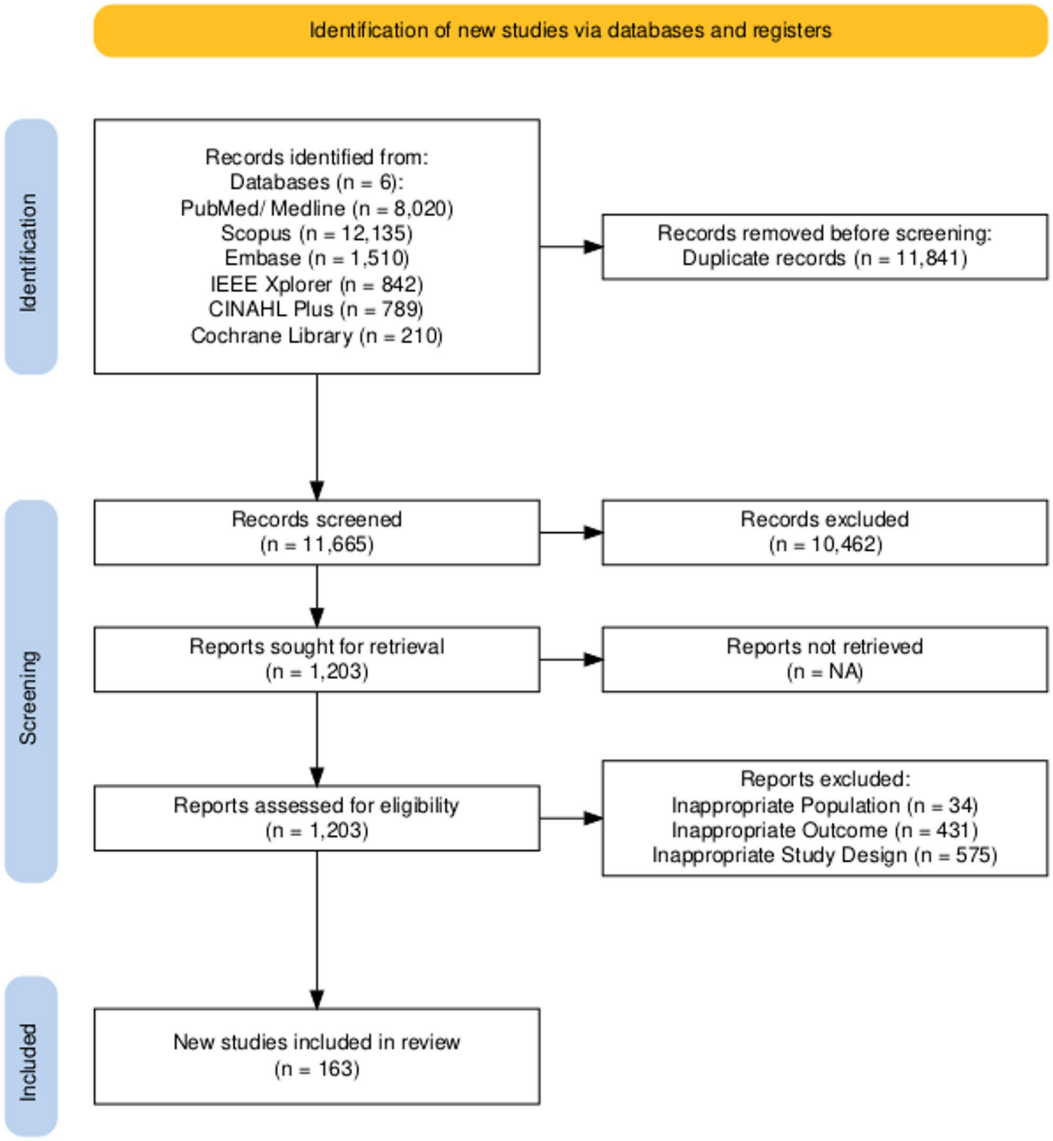
Flow chart of the methodology. The literature search was conducted using the following databases: PubMed/Medline, Embase, Scopus, IEEE Xplore and CINAHL. Of the initial 23,506 articles, 11,665 unique entries remained after deduplication. Upon screening the titles and abstracts, a further 10,462 articles were excluded. The remaining 1,203 articles were retrieved in full due to institutional agreements with publishers and underwent full-text scrutiny. Of these, 34 focused on populations that were inappropriate for the study, as the majority or all of the cohort under study did not have central nervous system pathologies. A further 431 articles did not elaborate sufficiently on the therapeutic or diagnostic dimensions of AI-based biomarkers, and 575 articles did not satisfy the agreed study design criteria. Thus, the final review included 163 unique, fully eligible articles

The articles retrieved from the requisite databases were first deduplicated using EndNote 2025. The inclusion and exclusion criteria were then applied to the remaining unique articles. Four authors (S.H.A., V.S., A.S. and P.A.N.) independently reviewed the articles at the title and abstract level, and then at full-text level, to ensure accordance with the eligibility criteria. In cases of disagreement regarding inclusion, the senior author (A.A.W.) consulted all the reviewing authors but reserved the right to make the final decision. The ‘References’ sections of the selected articles were also manually screened to identify any articles that might have been missed by our strategy. This process is illustrated in a PRISMA (Preferred Reporting Items for Systematic Reviews and Meta-Analyses) flowchart (Fig. 1). Out of an initial 23,506 articles, 11,665 unique entries remained after deduplication. Upon screening at the title and abstract level, a further 10,462 articles were excluded. The remaining 1,203 articles were retrieved in full due to institutional agreements with publishers and underwent full-text scrutiny. Of these, 34 were on inappropriate populations, where the majority or all of the cohort under study did not have CNS pathologies; 431 did not elaborate sufficiently on the therapeutic or diagnostic dimensions of AI-based biomarkers; and 575 did not satisfy the decided-upon study design criteria. Thus, a final 163 unique, fully eligible articles were included in the review.

## Biomarkers and biomarker discovery in neurological and neuroscience research

### Definition and classification of biomarkers in medicine

The term “biomarker” derives from “biological marker” and refers to a category of medical signs that function as objective indicators which can be measured accurately and reproducibly outside the patient [14]. In clinical and research contexts, biomarkers are defined as measurable characteristics that reflect normal biological processes, pathological alterations, or responses to therapeutic interventions [15–17].

Although various definitions exist, they largely overlap. For example, in a World Health Organization (WHO)-led joint programme on chemical safety, a biomarker was defined as “any substance, structure, or process that can be measured in the body or its products and that can influence or predict the occurrence, progression, or outcome of disease” [14]. This broad definition highlights the multifaceted role biomarkers play in modern medicine.

In practice, biomarkers can be used to follow the course of a disease, stage or grade its severity, guide treatment decisions, and monitor response or detect recurrence [16]. They should not, however, be confused with clinical outcome assessments (COAs). Biomarkers provide objective measures, such as blood glucose concentration, cerebrospinal fluid (CSF) protein levels, or neurofilament light chain (NfL), which has emerged as a promising biomarker of axonal injury in multiple sclerosis and other neurodegenerative conditions [18]. In contrast, COAs capture aspects of a condition that matter directly to patients, such as pain scores or quality-of-life questionnaires, and are often used to evaluate therapies. Because COAs rely on patient-reported or behaviour-based information, they may be shaped by factors such as judgment, motivation, or personal choice, and therefore reflect how a patient feels, functions, or survives [15, 19, 20].

Due to their varied definitions and applications, biomarkers can be categorised systematically according to their function or type, offering a structured approach to their role in clinical practice and biomedical research.

#### Classification according to function

Biomarkers can be grouped by clinical role into diagnostic, monitoring, pharmacodynamic/response, predictive, prognostic, susceptibility/risk, and safety biomarkers [15, 17]. Diagnostic biomarkers confirm the presence of disease and may also help classify subtypes, supporting personalised medicine. One example is CSF tau protein and amyloid-β42, which are used in the diagnosis of Alzheimer’s disease [21]. Prognostic biomarkers, in contrast, provide information about how a disease is likely to progress or the chance of recurrence. Serum creatinine is often measured in spinal muscular atrophy, where reduced levels reflect loss of muscle mass and are associated with poorer motor function [21]. Predictive biomarkers indicate the likelihood of responding to a particular therapy and help guide treatment selection [16, 22]. In neuro-oncology, genetic alterations such as epidermal growth factor receptor (EGFR) and BRAF influence treatment response and play a key role in tumour biology [22].

Monitoring biomarkers are assessed repeatedly over time to track disease status and evaluate responses to therapy. Neurofilament proteins, for example, are increasingly used as monitoring biomarkers in neurological disorders such as multiple sclerosis, amyotrophic lateral sclerosis (ALS), traumatic brain injury (TBI) and Parkinson’s disease (PD) [6]. Pharmacodynamic/response biomarkers provide valuable insights into the biological effects of therapies, including proof of mechanism, identification of response or resistance, and support in drug development through sample stratification [23–25]. Susceptibility/risk biomarkers identify individuals with an increased likelihood of developing disease prior to symptom onset, improving understanding of disease pathophysiology and enabling preventative strategies [26–28]. For instance, gait-related biomarkers such as asymmetry and variability have been used to evaluate susceptibility to PD [27]. Finally, safety biomarkers indicate the presence or extent of toxicity before or after exposure to therapeutic or environmental factors [29].

Taken together, these functional classifications highlight the versatility of biomarkers in modern medicine, ranging from early detection and risk assessment to treatment monitoring and safety evaluation.

#### Classification according to nature

Biomarkers can also be categorised into four broad types: molecular, physiological, histological, and radiographic [30]. Molecular biomarkers refer to molecules such as proteins, metabolites and deoxyribonucleic acid (DNA), and reflect genetic and metabolic physiology. They can be measured directly from serum, plasma, CSF, or tissue samples. Histologic biomarkers describe cellular and tissue characteristics identified through biopsy and microscopic analysis and are particularly useful in oncology for diagnosis, staging, prognosis, and guiding management [31].

Physiological biomarkers refer to indicators of the body’s functional status, including blood pressure, heart rate, electroencephalography (EEG) and electrocardiogram, which can be obtained rapidly and non-invasively [32]. Radiographic biomarkers are derived from imaging modalities such as X-ray, ultrasound, computed tomography (CT) and MRI, providing non-invasive structural and diagnostic insight [33]. Imaging biomarkers are a subtype of this category and describe precise, quantifiable features such as lesion size, morphology, and contrast uptake [34]. Table 1 provides a summary of the classification of biomarkers in medicine.

**Table 1:** Classification and functions of biomarkers in medicine [30–34]

Biomarker classification	Classification subtype	Functions
1. Biomarkers according function	Diagnostic	Identify specific pathologies and support the principles of personalised medicine
	Monitoring	Repeatedly assessed over time to track disease status, predict outcomes, and evaluate responses to therapy
	Prognostic	Offer valuable insights into the biological effects of therapeutic agents, providing key information, including proof of mechanism and concept, as well as identification of response and resistance mechanisms
	Pharmacological	Provide information on the likely progression of a disease or the risk of recurrence
	Predictive	Provide information on the likelihood of achieving a response to a particular treatment, as this will help patients to anticipate therapeutic responses to specific drugs and guide treatment selection
	Susceptibility/risk biomarkers	It identifies individuals with an increased likelihood of developing a disease before clinical symptoms appear In the field of neurology, this early identification improves our understanding of disease pathophysiology and enables us to implement preventative strategies
	Safety biomarkers	Measured before or after exposure to a therapeutic intervention or environmental factor, in order to indicate the presence or extent of toxicity as an adverse effect
2. Biomarkers according to nature	Molecular	They include DNA, RNA, proteins and metabolites that reflect genetic, metabolic or physiological processes. These can be measured directly in biological samples, such as blood, plasma, CSF or tissue biopsies These biomarkers provide insight into genetic predispositions and disease mechanisms and play a key role in disease diagnosis
	Histologic	They define the structural and cellular characteristics of tissues that are commonly assessed using biopsies and further microscopic analysis These techniques are used to highlight cellular features and proteins in tissues and are widely used in oncology for tumour classification, grading and staging
	Radiographic	These morphologic features are detectable on scans These features can be analysed using various imaging modalities, including X-ray, ultrasound, CT and MRI These modalities enable the rapid, versatile, non-invasive analysis of various body structures and tissues, and are already relied upon for diagnosis, treatment guidance and disease monitoring across a wide range of disorders
	Physiological	Physiological biomarkers are indicators of the body’s physiological status These include blood pressure, heart rate, EEG and ECG. They can be measured rapidly and non-invasively, with minimal observer training required They provide vital insight into systemic function and are routinely used in clinical practice to screen hospitalised patients for early deterioration

DNA, deoxyribonucleic acid; RNA, ribonucleic acid; CSF, cerebrospinal fluid; EEG, electroencephalography; ECG, electrocardiography; MRI, magnetic resonance imaging; CT, computed tomography

### Current biomarkers associated with neurological conditions

The diagnostic and prognostic utility of biomarkers in various neurological disorders has attracted increasing attention. In neurodegenerative disorders (NDDs), for instance, amyloid-β peptides and phosphorylated tau are well-established biomarkers reflecting neuronal and glial degeneration in AD. Misfolded alpha-synuclein that aggregates into Lewy bodies is a diagnostic hallmark of PD, while the neuron-specific axonal protein NfL is increasingly being used to track disease progression [35, 36]. In neurovascular cases such as stroke and TBI, glial fibrillary acidic protein (GFAP), which is released during astroglial injury, is a valuable marker of acute CNS damage. Matrix metalloproteinase-9 (MMP-9), which degrades extracellular matrix components, contributes to infarct progression in both ischaemic and haemorrhagic stroke and provides insight into the prognosis of secondary injury and recovery [37].

Neuro-oncological biomarkers also include O-6-methylguanine-deoxyribonucleic acid methyltransferase (MGMT) promoter methylation, co-deletion of 1p/19q and isocitrate dehydrogenase (IDH) 1/2 mutations. These molecular alterations aid the diagnosis and classification of glioblastomas and oligodendrogliomas according to the WHO grading criteria [38–40]. Furthermore, in epilepsy and seizure disorders, biomarkers such as the neuronal protein S100B, glial fibrillary acidic protein (GFAP) and neuron-specific enolase (NSE) have demonstrated diagnostic and prognostic value. S100B has been reported in both adult and paediatric temporal lobe epilepsy and has shown a stronger association in females. GFAP, due to its relatively long half-life, can be detected in patients experiencing seizures, including post-stroke cases. Meanwhile, elevated NSE levels correlate with seizure episodes [41].

Electrophysiological biomarkers are another important category, particularly in the case of neurodevelopmental disorders. Techniques such as EEG, EMG, evoked potentials and event-related potentials offer valuable insights into brain function and can be used to diagnose conditions such as autism [42].

### Conventional biomarker discovery/detection methods

Biomarker discovery typically starts with 'omic' technologies, which use methods such as mass spectrometry, next-generation sequencing and microarrays. Genomics focuses on sequencing, whereas transcriptomics techniques, including DNA microarrays and gene expression profiling analyse ribonucleic acid (RNA) in order to characterise the host's response to disease [43, 44]. Proteomics uses mass spectrometry and protein chips to identify proteins in biological samples that may indicate disease or environmental exposure. Metabolomics, on the other hand, analyses small molecules using methods such as mass spectrometry and nuclear magnetic resonance. Together, these methods are essential for biomarker discovery, helping to identify genetic predispositions and elucidate host responses to disease [44–46]. The conventional biomarker discovery methods have been summarised in Table 2.

**Table 2:** Conventional biomarker discovery methods and their functions [43–46]

Conventional biomarker discovery method	Description
Genomics	Identifies genetic variants ranging from single nucleotide substitutions to large scale genome rearrangements Genomic biomarkers help explain phenotype variability, disease susceptibility and therapeutic response enabling personalised treatment
Proteomics	Profiles proteins expressed in cells and tissues using mass spectrometry, protein microarrays and electrophoresis Analyses protein expression, interactions and post-translational modifications Enables study of cellular responses and functions, disease mechanisms and biomarker discovery
Metabolomics	Describes metabolite analysis of a specimen using mass spectrometry and nuclear magnetic resonance Reveals metabolic changes which reflect normal physiology as well as cellular responses to exposures and disease states

### Limitations in the conventional biomarker discovery methods and the need for AI

Despite the substantial advances achieved through conventional biomarker discovery approaches, these methodologies are increasingly constrained by practical, technical, and conceptual limitations. Traditional biomarker pipelines are often expensive, time-consuming, and labour-intensive, requiring extensive experimental validation and iterative hypothesis testing [43]. Moreover, they typically rely on predefined analytical rules and handcrafted feature selection, necessitating significant human expertise and introducing subjectivity into the discovery process. This dependence not only slows the pace of biomarker identification but also limits scalability, particularly in the context of rapidly expanding high-throughput datasets [43, 47].

A further critical limitation of conventional biomarkers lies in their suboptimal sensitivity and specificity, which are essential determinants of diagnostic and prognostic accuracy. Many established biomarkers demonstrate limited robustness across heterogeneous patient populations and disease subtypes, thereby restricting their clinical utility and impeding translation into personalised therapeutic strategies [43]. These shortcomings are particularly pronounced in complex, multifactorial diseases, where single or narrowly defined biomarkers often fail to capture underlying biological heterogeneity.

The emergence of large-scale omics technologies has further exposed the inadequacy of traditional analytical frameworks. High-dimensional datasets generated from genomics, transcriptomics, proteomics, and metabolomics are characterised by complex nonlinear relationships, skewed data distributions, and frequent outliers, all of which challenge conventional statistical approaches [47]. As a result, classical methods often struggle to extract meaningful patterns, integrate multimodal data, or generalise findings beyond narrowly defined cohorts.

AI, particularly ML and DL techniques, offers a compelling solution to these challenges. AI-driven approaches can rapidly interrogate vast, complex datasets, identify subtle and non-intuitive patterns, and construct highly accurate predictive models without reliance on rigid, predefined assumptions. These capabilities substantially enhance biomarker discovery pipelines, enabling improved disease diagnosis, prognosis, and therapeutic stratification, thereby advancing the goals of precision medicine [11, 43, 48].

Notably, AI has demonstrated exceptional performance in domains such as histopathological image analysis, where deep learning models can extract high-resolution spatial and morphological features that exceed the discriminatory capacity of many traditional techniques. This has led to marked improvements in diagnostic sensitivity and specificity, directly addressing longstanding limitations of conventional biomarker approaches. Furthermore, ML algorithms are inherently well suited to handling high-dimensional, multimodal biological data, facilitating the integration of diverse molecular, clinical, and imaging datasets into unified analytical frameworks [47, 49].

In summary, while conventional biomarker discovery methods have played a foundational role in biomedical research, their limitations have become increasingly apparent in the era of big data and systems biology. AI-based methodologies offer a transformative paradigm, providing the computational scalability, analytical flexibility, and integrative capacity required to fully exploit complex biological data and to develop robust, clinically actionable biomarkers tailored to individual patient profiles. The advantages of AI-based biomarkers over conventional technologies have been summarised in Table 3.

**Table 3:** Advantages of AI-based biomarkers over conventional technologies

Dimension	Conventional biomarker discovery	AI-based biomarker discovery
Analytical framework [43, 47]	Rule-based, hypothesis-driven, relies on predefined statistical models	Data-driven, adaptive learning using ML/DL without rigid assumptions
Scalability [47, 49]	Limited scalability; performance declines with increasing data dimensionality	Highly scalable; designed to process large, high-dimensional datasets
Time and cost efficiency [11, 43]	Time-consuming, labour-intensive, and costly due to iterative validation	Faster discovery pipelines with reduced manual intervention
Sensitivity and specificity [43, 48]	Often suboptimal; limited robustness across heterogeneous populations	Enhanced sensitivity and specificity through pattern recognition
Handling data complexity [47]	Struggles with nonlinearity, skewed distributions, and outliers	Effectively models nonlinear relationships and noisy data
Multimodal data integration [48, 49]	Limited ability to integrate multi-omics and imaging data	Seamless integration of multi-omics, imaging, and clinical data
Clinical translation [11, 43]	Slower translation to personalised medicine	Strong alignment with precision and personalised medicine

AI, artificial intelligence; ML, machine learning; DL, deep learning

## Accelerating diagnostics, drug discovery and personalising treatment through AI-based biomarkers and biomarker discovery for neurological conditions

### Neurovascular diseases

#### Stroke

Stroke is primarily diagnosed using traditional imaging strategies, such as non-contrast CT (NCCT) and MRI, which have yielded excellent results for many years and have helped to develop effective treatment strategies. However, these neuroimaging strategies can sometimes lead to diagnostic uncertainty, particularly in cases involving subtle or incidental findings. For example, CT unfortunately lacks sensitivity in diagnosing acute ischaemic stroke (AIS), and while MRI is an excellent imaging modality for detecting ischaemic stroke, it is less accessible, more expensive, and more time-consuming, which makes it challenging to use in such situations. Furthermore, determining lesion age and predicting the progression of acute ischaemic lesions is pivotal for clinical practice, as the management and prognosis of stroke depend heavily on these factors [50]. There is a growing body of evidence demonstrating the utility of AI in enhancing the prediction, diagnosis, risk stratification and prognostication of stroke, thereby addressing several key gaps in current practice.

##### Predicting stroke and its outcomes

One of the most important applications of AI-driven biomarker analysis in the context of stroke is its ability to predict outcomes. A large, multicentre study demonstrated that DL imaging analysis using the CNNdeep model in combination with traditional risk scores for predicting three-month outcomes was associated with significant improvements in prediction accuracy. The study concluded that DL enhances the predictive ability of the scores currently in use [51].

Another interesting study developed models to predict the functional effects on patients after stroke, determining outcomes using the modified Rankin Scale after three months, categorised as good (score > 2) or poor (score ≤ 2). [52]. Three approaches were trialled across 743 patients: some were given only the NCCT scan; others were provided with additional patient details; and the remainder were given both. As expected, 'image-only' models underperformed, reflecting the fact that admission CTs in the context of ischaemic stroke are often relatively unremarkable and thus unable to fully capture the true impact of stroke. [52]. By contrast, hybrid models that incorporated both raw imaging biomarkers (specifically the Alberta Stroke Program Early CT Score (ASPECTS) score) and patient factors performed better. Notably, the best performance was not achieved by complex DL models, but by a simple logistic regression model (LR 5vars) using just five predictors: age, National Institutes of Health Stroke Scale score, glucose level, ASPECTS score and computed tomography angiography (CTA)-derived occlusion. [52].

A further study by Yang et al. (2024) investigated the ability of AI models to make similar predictions using MRI scans. This large-scale study (n = 3338) involved training a DL model on MRI interpretation and comparing it with five recognised clinical scores. With imaging data alone, the model's outcomes were comparable to the clinical scores; however, when both were combined, improved predictive performance was observed. [53] This suggests that AI models using imaging data alone can produce results that are comparable to those of validated clinical scores and that AI's greatest contribution in this context may be to augment existing risk stratification scores.

Retinal imaging has emerged as a powerful tool, with Retinal Biomarker Extraction and Stroke Risk Assessment models trained on large datasets such as the United Kingdom Biobank. By analysing retinal microvasculature features, including vessel diameter, bifurcation angles, tortuosity, and signs of diabetic retinopathy, these models predicted stroke risk and timing with area under the receiver operating curve (AUROC) values of 0.83–0.93, significantly outperforming traditional systems such as Framingham [54].

Transcriptomic analyses have uncovered novel inflammatory biomarkers linked to ischemic stroke. DL methods identified dysregulated inflammation-related genes such as *AHR*, *OSM*, and *NMUR1*, achieving area under the curve (AUC) values above 0.9 and correlating these changes with immune cell shifts, including elevated CD4 + T-cells and neutrophils, and reduced CD8 + T-cells and natural killer cells [55]. Additional work highlighted *RNF13*, *VASP*, and *CD163* genes, which achieved AUC values of 0.97–1.0, further demonstrating AI’s ability to move beyond known biomarkers and reveal new diagnostic targets [56].

Beyond imaging and transcriptomics, blood-based biomarkers analysed by AI also show clinical promise. Models trained on routine laboratory data, including blood counts, coagulation profiles, lipid levels, and cardiac enzymes, discriminated between cardioembolic, large-artery atherosclerotic, and small-vessel stroke subtypes with accuracy comparable to emergency department clinicians. This approach may enable rapid classification of AIS etiology, crucial for tailoring treatment strategies [57].

##### Lesion age estimation

It is possible to estimate the age of an ischemic lesion on neuroimaging, and the most accurate NCCT method currently available is Net Water Uptake (NWU). However, this approach is imperfect. A recent multi-centre study applied the DL model CNN-R to NCCTH’s; it was first trained to age lesions on CT scans before analysing unlabelled scans, and predicted lesion age with nearly twice the accuracy of NWU [50]. The CNN-R’s estimates were also more strongly correlated with ischemic core-to-penumbra ratio than NWU and clock time, and it predicted early infarct expansion better than conventional methods. Importantly, validation across multiple centres and scanner types showed consistent success, underscoring how AI models not only improve diagnostic accuracy but also extract biomarkers that reflect stroke biology and prognosis [50].

##### Microinfarct diagnosis

AI-models were able to identify microscopic ischemic lesions on NCCT that were imperceptible to radiologists [58]. This involved over a thousand patients with suspected AIS, who had NCCT’s reported as normal but later underwent diffusion-weighted MRI, which detected microscopic AIS in some cases. The AI models then analysed these NCCT’s and reliably detected a high proportion of lesions that radiologists had missed across external cohorts [58]. A DL model further validated these results by analysing over 1,000 ‘unremarkable’ NCCT scans, achieving an AUC 12% higher than senior neuroradiologists and improving their accuracy by 25–30% when assisted [59].

Another development was the DL system ‘DeepRETStroke’, designed to diagnose silent brain infarctions (SBIs). SBIs affect about 20% of the global population and are strong predictors of future stroke, yet widespread screening is not feasible due to cost, radiation, and limited sensitivity. DeepRETStroke addressed this by analysing retinal photographs as a ‘window to the brain.’ Trained on nearly 900,000 images and validated across diverse populations, it achieved an AUC of 0.901 for predicting SBIs and 0.769 for future stroke [60]. This approach offers a scalable, non-invasive screening tool with the potential to transform stroke prevention strategies worldwide. Together, these findings highlight the potential of AI in detecting microinfarcts and enabling earlier, more accurate stroke diagnosis.

#### Intracranial aneurysms

##### Diagnosing aneurysms

DL has been shown to be useful in various other areas of neurovascular disorders, most notably in the case of intracranial aneurysms. This is a vital area of research as aneurysms are highly prevalent, affecting around 3–4% of the general population. Furthermore, aneurysm-related subarachnoid haemorrhages carry a 44% mortality rate and a 20% rate of permanent disability among survivors [61]. Early and accurate detection of aneurysms allows time for risk stratification and timely intervention to prevent rupture, thus significantly improving prognosis. Aneurysm detection requires CT angiography, and the accuracy of this investigation depends on factors including aneurysm size, location, image quality, and radiologist experience. Furthermore, accurate reporting demands time, focus, and proficiency, all of which can be compromised by human factors such as fatigue. Given the lethality of aneurysms, it is essential to optimise early detection and risk stratification, challenges that AI can significantly ease. Some studies have criticised the accuracy of CTA, stating that the rate of missed aneurysms may be as high as 21.6% [62] and that this rate is particularly high for small aneurysms [63]. Although the risk of rupture is much smaller for aneurysms measuring less than 4 mm (0.36%), approximately 35% of aneurysms are less than 5 mm. Thus, aneurysms within this range are still important not to miss. [64].

A recent study developed DL models (the Aneurysm Detection Model and the Aneurysm Segmentation Model) that can detect cerebral aneurysms. The study involved almost 4,000 patients across 11 clinical centres and aimed to assess the performance of the models independently and alongside junior and senior radiologists. Using the DL resulted in a significant increase in interpretation accuracy amongst both groups of radiologists. Furthermore, the use of DL was associated with a 37% reduction in image interpretation time [61]. The study concluded that, when radiologists utilised DL, the rate of total aneurysms missed during CTA reporting was as low as 1%. This highlights how AI can substantially reduce human error and increase reporting efficiency [61].

Rodríguez-Regente et al. (2014) found that, when used alone, AI could detect aneurysms with 91.2% sensitivity (95% CI 82.2–95.8%), almost matching the sensitivities published for CTA interpretations by radiologists (95% CI 92.8–100%) [65]. Other analyses report that AI models have demonstrated superior diagnostic abilities to those of radiologists and neurosurgeons, with 100% sensitivity for aneurysms > 5 mm and 98.6% for those > 3 mm, as well as a 99% specificity rate. This suggests that AI could reliably reduce the human workload to a great degree [65–67].

Although the detection of aneurysms on CTA is very efficient, it still poses a risk as the size, location and morphology of the aneurysm strongly influence the prognosis. Currently, the accuracy of these imaging biomarkers is imperfect as analysis depends on image quality and the expertise of the radiologist, leaving room for human error. AI models have emerged as reliable tools for assessing these biomarkers, improving sensitivity in the detection of small aneurysms and reducing subjective variability.

##### Differentiating between stable and unstable aneurysms

Another area of concern is distinguishing between stable and unstable aneurysms, as this, along with size, is one of the main factors that determine the subsequent management offered to patients (conservative versus surgical). Assessing stability status adds another layer of complexity to interpreting aneurysms, with potentially catastrophic consequences if misjudged. Furthermore, procedures such as neurosurgical and endovascular clipping carry a 5% risk of complications and must therefore be reserved for necessary cases. As such, this is yet another area in which researchers are eager to appraise the utility of AI [68]. Although risk stratification aids such as the ELAPSS score currently exist, concerns remain regarding their low sensitivity. [63].

Such scores currently have limited performance as they mainly rely on patient characteristics and basic morphological features. In contrast, AI-based models incorporate more complex data and have been shown to outperform these scores in several studies. Beyond morphology, research has revealed that additional factors, such as abnormal intra-aneurysmal blood flow and weak aneurysmal walls, are key metrics that determine aneurysm stability [69]. Flow-related features such as high and low abnormal wall shear stress, unstable vortical flow patterns, and small impingement sites are strongly associated with instability. Furthermore, inflammation plays a role, with unstable aneurysms exhibiting infiltration by macrophages and T cells, as well as increased production of cytokines such as TNF-α, IL-1 and MCP-1. Excess protease activity contributes further to this, leading to extracellular matrix breakdown and thus increased aneurysmal wall fragility. These features represent measurable biomarkers that AI systems could integrate to potentially enable the automated prediction of aneurysmal stability and thus rupture risk [70].

Current methods of assessing aneurysmal blood flow include computational fluid dynamics studies based on CT angiography imaging [68]. However, these techniques are based on the assumption of rigid boundaries due to the inability to accurately extract this information. This leads to an overestimation of intra-aneurysmal shear stress and limits the ability to capture patient-specific haemodynamics [70]. The emergence of more complex imaging techniques enables the measurement of these parameters. High-resolution MRI provides information on the aneurysm wall, such as enhancement and thickness, which reflect inflammation and strength, while 4D-MRI allows the evaluation of haemodynamics. However, these measures are subjective as they require classification (AWE classification) by the interpreting radiologist and the data is summarised as single numbers (contrast ratio and average wall shear stress). Therefore, although the relevant data can be extracted from the available imaging techniques, integrating them to maximise predictions remains challenging. Peng et al. (2024) developed a hybrid model consisting of a 4D-Flow-LR and MicroAB-Net model and proposed it as a solution to this problem. These systems can analyse various modalities, extracting the necessary complex data including that which radiologists are unable to visualise, such as the microtextures of the aneurysm wall reflecting inflammation and combining this information to provide more detailed predictions. [68] The results concluded that the model's performance was significantly superior to the currently used PHASES and ELAPSS scores (0.817 vs. 0.725 and 0.774, respectively) [68].

#### Cerebral venous thrombosis

Cerebral venous thrombosis (CVT) is a rare subtype of stroke caused by clot formation in the brain’s venous sinuses or cerebral veins, leading to venous outflow obstruction and increased intracranial pressure [71]. Diagnosis is challenging because clinical features are often non-specific and can mimic conditions such as migraine or idiopathic intracranial hypertension. The potential use of biomarkers combined with AI-assisted approaches has therefore become a focus for improving diagnostic accuracy in CVT [71].

Several inflammatory and haemostatic markers have been investigated as potential biomarkers for CVT, including the systemic immune inflammatory index (SII), platelet-to-lymphocyte ratio (PLR) and neutrophil-to-lymphocyte ratio (NLR), all of which are derived from routine blood counts and reflect systemic inflammatory and thrombotic processes [72]. NLR is already established as a marker in cardiovascular disease and has been proposed as a candidate in CVT [Zhou et al., 2024]. MMP-9, a protease involved in extracellular matrix degradation and disruption of tight junctions, has also been linked to the pathophysiology of CVT [72].

ML trained on these inflammatory indices have shown strong diagnostic performance. An ANN using SII and PLR inputs achieved an average ROC of 0.94, demonstrating excellent sensitivity and reliability for CVT classification [72]. DL approaches have also been applied to imaging datasets. A large study of nearly 400 patients, including 294 with confirmed CVT, evaluated multiple DL models against radiologist interpretation of MRI scans [73]. The multisequence multitask DL (MSMT-DL) model achieved 96% sensitivity and 88% specificity, significantly exceeding the diagnostic sensitivity of radiologists (72–78%) while maintaining comparable specificity [73].

The MSMT-DL model detected 30–44% more thrombosed venous segments than radiologists, confirming that AI can extract subtle imaging biomarkers not identified by human observers [73]. The superior performance of this model was attributed to its integration of T1-weighted, T2-weighted and fluid-attenuated inversion recovery sequences, which provided complementary information and enabled comprehensive thrombus characterisation [73].

**Fig. 2:**
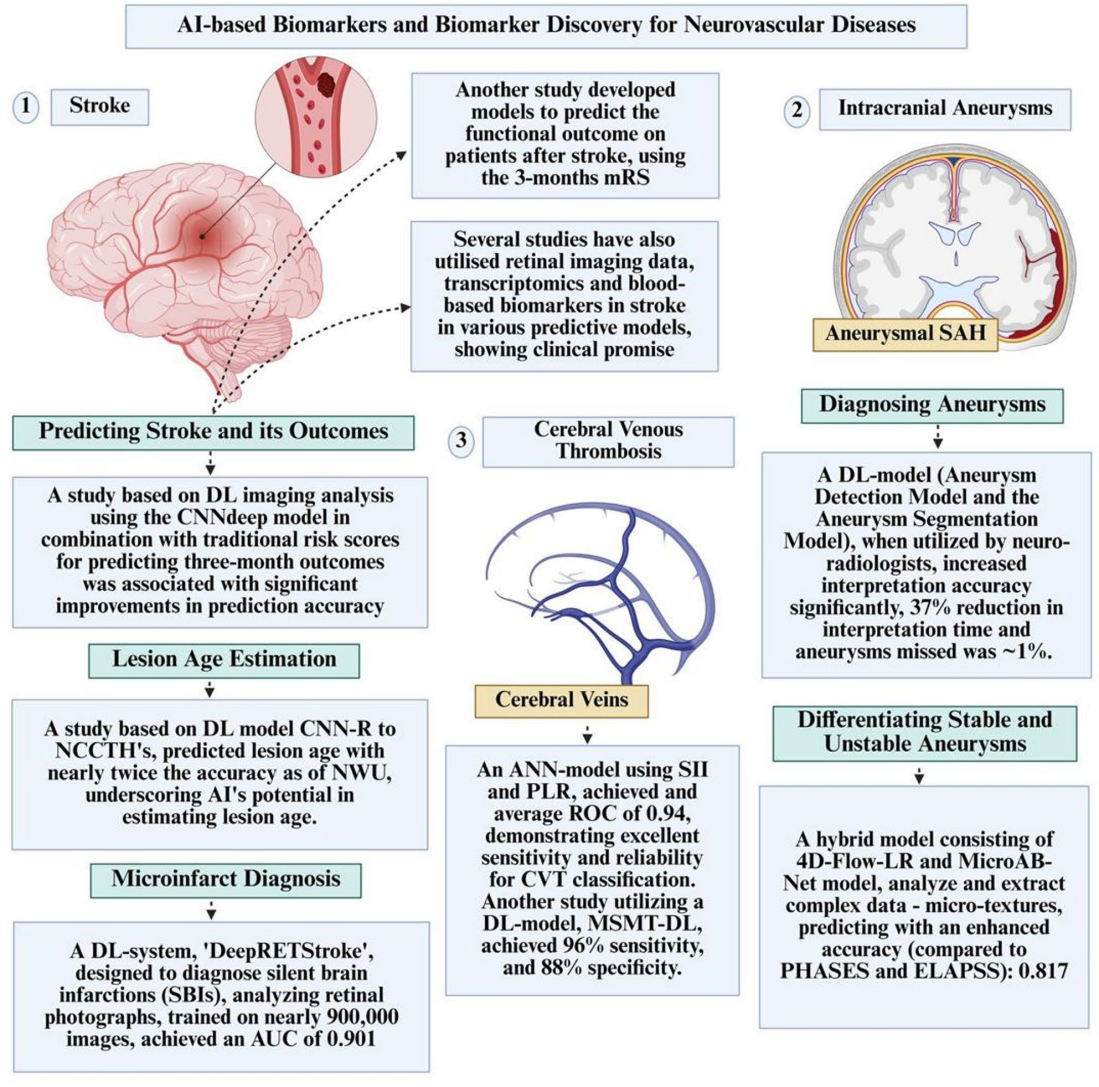
The role of AI-based biomarkers and biomarker discovery in neurovascular diseases. AI, artificial intelligence; DL, deep learning; CNNdeep, convolutional neural network (deep learning model); CNN-R, convolutional neural network-regression; NCCTH, non-contrast computed tomography of the head; mRS, modified rankin scale; NWU, net water uptake, SBI, silent brain infarction; ANN, artificial neural network, PLR, positive likelihood ratio; SII, systemic immune-inflammation index; ROC, receiver operating characteristic; CVT, cerebral venous thrombosis; MSMT-DL, multisequence multitask deep learning; SAH, subarachnoid hemorrhage. Created with Biorender.com

Evidence across multiple neurovascular disorders, including ischaemic stroke, intracranial aneurysms and CVT, consistently supports the use of AI-driven biomarker models to enhance diagnostic sensitivity, improve prognostication and inform tailored decision-making [71–73]. These systems frequently equal or surpass expert human performance, with advantages most pronounced in multimodal data integration [73]. Future research must prioritise multicentre validation and reproducibility to enable safe clinical translation and adoption. Figure 2 is a visual representation of the role of AI-based biomarkers and biomarker discovery in neurovascular diseases.

### Neurodegenerative diseases

#### AI for biomarker discovery in NDDs

Traditional discovery methods such as enzyme-linked immunosorbent assay, immunohistochemistry, volumetric MRI and diffusion tensor imaging have advanced the field but remain reductionist, often yielding inconsistent findings and requiring 5–10 years for validation [74, 75]. AI transforms this process by analysing genomics, transcriptomics, proteomics, metabolomics, neuroimaging and digital phenotyping simultaneously, uncovering complex interactions that better reflect NDD biology [76].

In AD, AI applied to plasma proteomics has identified lipidomic and inflammatory signatures that predict risk years before symptoms with accuracies above 85%, surpassing CSF amyloid assays that rarely exceed 70% sensitivity [76]. AI-enhanced structural MRI detects subtle hippocampal shape changes and cortical thinning trajectories associated with conversion from mild cognitive impairment (MCI) to AD, patterns invisible to manual or linear analyses [77]. Models that integrate retinal imaging with blood biomarkers now rival positron emission tomograĥy (PET) accuracy, pointing to scalable and non-invasive diagnostic approaches [78]. In ALS, AI applied to transcriptomic and proteomic data has revealed RNA-binding proteins and inflammatory mediators as prognostic markers, outperforming clinician-based scales and capturing longitudinal biomarker changes across disease stages [79, 80].

At the molecular level, AI-integrated plasmonic infrared nanosensors enable detection of misfolded proteins, including α-synuclein and tau, at femtomolar concentrations, far surpassing the sensitivity of conventional immunoassays [81]. Similarly, AI-powered radiogenomic frameworks, particularly CNNs, uncover complex, non-linear relationships between imaging-derived phenotypes and underlying genetic mutations, revealing associations that are inaccessible through traditional correlation analyses [82]. These enable the integration of imaging phenotypes with underlying genetic mutations, uncovering complex, non-linear associations that are invisible to conventional correlation analyses [82]. This capability dramatically enhances biomarker discovery in NDDs by linking structural and functional changes observed in neuroimaging with molecular and genetic alterations. As a result, researchers can identify predictive, multimodal biomarker signatures that reflect the systemic pathophysiology of disorders such as AD, PD, and ALS, providing deeper insight into disease mechanisms and facilitating early detection, patient stratification, and personalised intervention strategies.

Studies demonstrate a marked surge in AI-driven biomarker research since 2000, with a pronounced acceleration after 2015, driven by advances in DL algorithms and the availability of large, high-quality datasets, highlighting the stark contrast with the incremental progress characteristic of classical, hypothesis-driven biomarker discovery [83, 84]. In the context of NDDs, this trend reflects a critical shift: AI enables the identification of complex, multimodal biomarker signatures that were previously inaccessible due to the limitations of traditional hypothesis-driven approaches. Unlike conventional methods, which often rely on single markers or small datasets and yield incremental findings, AI-powered analyses integrate molecular, imaging, and clinical data to uncover dynamic patterns and interactions across disease stages. This surge in research therefore represents not only technological progress but also a transformative step toward earlier diagnosis, more accurate prognostication, and discovery of novel therapeutic targets in NDDs.

Collectively, these AI-enabled approaches redefine biomarker discovery in NDDs by shifting from reductionist, single-marker strategies to systems-level analyses that capture dynamic biomarker constellations and trajectories across disease stages, facilitating earlier diagnosis, improved prognostication, and identification of novel therapeutic targets [76, 85].

#### AI biomarkers enhancing NDD prediction and diagnosis

Conventional diagnostic pathways based on clinical scales, single biomarkers and linear models cannot capture the high-dimensional, non-linear biology of AD, other dementias and ALS [83, 86]. Traditional diagnostic pipelines rely on clinical scales, univariate biomarker assays, and hypothesis-driven statistical models, such as linear regression or Cox proportional hazards models [80]. While these approaches have provided important insights, they are limited by their inability to capture the non-linear, high-dimensional interactions that characterize disease progression in various NDDs [83]. Multimodal AI models integrate molecular, imaging and genetic inputs, improving prediction and stratification [80, 87].

In AD, AI models combining CSF amyloid and tau levels with PET imaging and polygenic risk scores predict conversion from MCI to AD with 85–90% accuracy, compared to 70–75% with regression-based methods. These models also estimate time-to-conversion, a capability not possible with conventional biomarkers [88]. In ALS, DL applied to transcriptomic and neurophysiological datasets distinguishes rapid from slow progressors, a key factor for prognosis and trial design that univariate approaches cannot achieve [79, 89]. AI reduces false negatives in early disease detection by uncovering latent, cross-domain associations missed by conventional approaches [76, 87]. Beyond accuracy, AI models also provide time-to-conversion predictions, enabling clinicians to identify high-risk individuals for early intervention, an advance not achievable using traditional biomarkers alone [76, 77].

Similarly, in ALS, DL applied to transcriptomic and neurophysiological datasets has yielded biomarker panels that accurately distinguish rapid from slow disease progressors, an achievement beyond traditional univariate biomarker analyses, which fail to capture the combinatorial effects of genetic and physiological variables [79]. These predictive models hold particular clinical relevance for prognosis and therapeutic trial design, where heterogeneity in progression has historically undermined statistical power [79].

Comparative analysis consistently highlights the superiority of AI approaches over standard biomarker pipelines. Conventional biomarker discovery remains hypothesis-driven, reductionist, and largely univariate, focusing on single-pathway associations that inadequately reflect the systemic complexity of NDDs [80, 83]. By contrast, AI leverages pattern recognition, clustering, and predictive analytics to uncover latent associations across heterogeneous biological domains, integrating molecular, imaging, and digital biomarkers into coherent predictive frameworks [80, 87]. This paradigm shift enhances both sensitivity and specificity, while reducing the risk of false negatives in early disease detection [76].

These advances have direct clinical utility. AI-based stratification reduces trial attrition rates by about 30% by enrolling homogeneous cohorts [90]. On the other hand, multimodal prognostic models improve outcome predictions by 15–20% compared with standard measures. This supports earlier and more personalised interventions [91].

#### Enhancing drug discovery and personalising treatment for NDDs

Drug development in NDDs has long suffered from failure rates exceeding 90%, largely due to poor target validation, inadequate stratification and disease heterogeneity [76, 90]. AI biomarkers address these barriers by enabling rational cohort selection, adaptive trial designs and systematic identification of novel therapeutic targets [89, 91, 92].

Tools such as DeepDrug use biomarker profiles to propose drug combinations tailored to specific subgroups, accelerating development and reducing attrition [93]. In ALS, AI-guided focus on RNA-binding proteins and inflammatory cascades has revealed new therapeutic targets [79]. Computational studies show AI can shorten drug development timelines by nearly 50% and improve trial success rates, especially when combined with open-science, multi-cohort validation [94]. AI-derived biomarkers in NDDs enable truly personalized treatment paradigms. By integrating multimodal data, including neuroimaging, genetic risk variants, and biochemical signatures, AI predictive models can forecast patient-specific responses to disease-modifying therapies, inform precision dosing strategies, and support adaptive therapeutic adjustments [79].

**Fig. 3:**
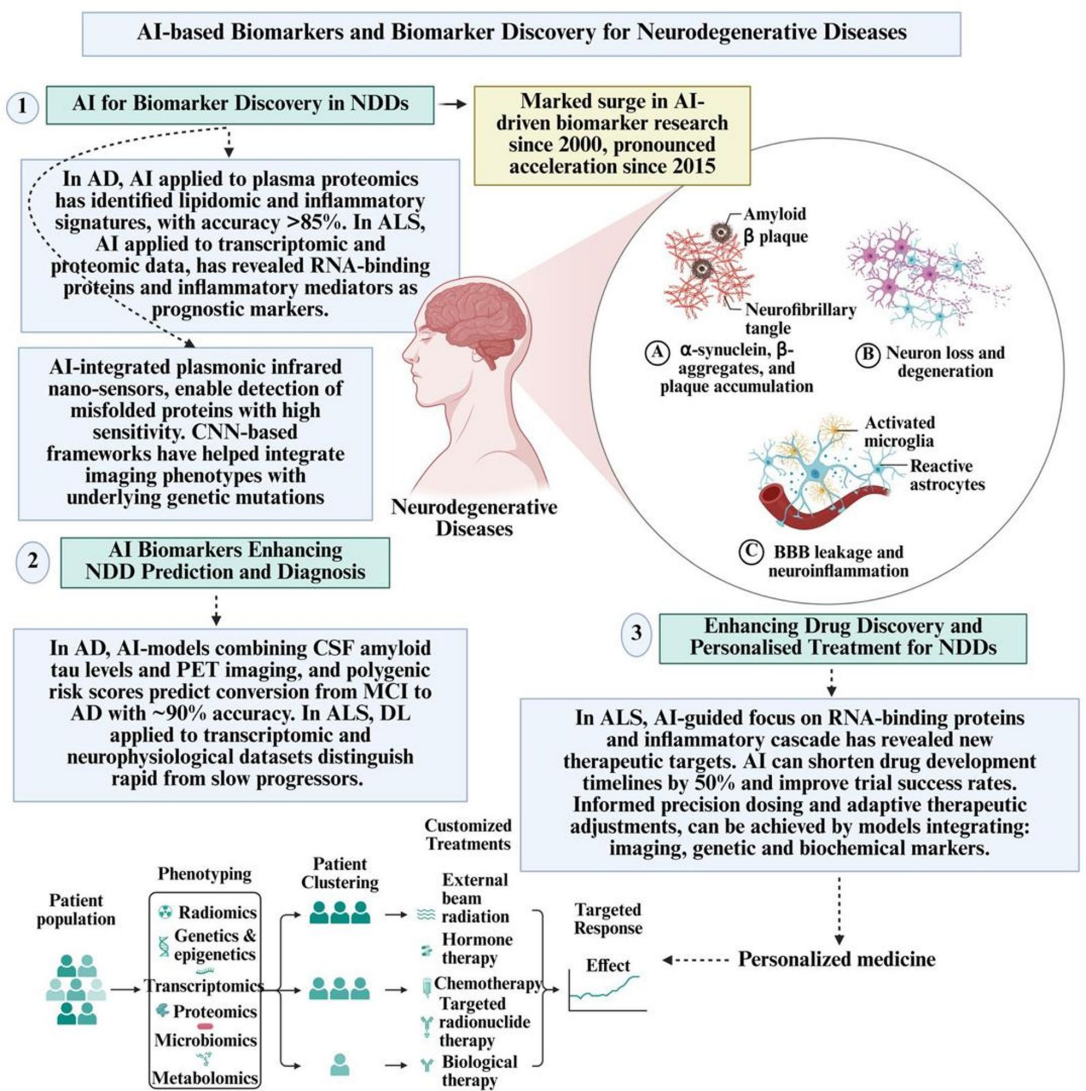
The role of AI-based biomarkers and biomarker discovery in neurodegenerative diseases. AI, artificial intelligence; NDD, neurodegerative disorder; ALS, amyotrophic lateral sclerosis; RNA, ribonucleic acid; CNN, convolutional neural network; CSF, cerebrospinal fluid; PET, positron emission tomography; MCI, mild cognitive impairment; AD, Alzheimer’s disease; DL, deep learning. Created with Biorender.com

Beyond accelerating drug discovery, AI-based biomarkers are central to advancing personalised treatment paradigms in NDDs. Predictive models integrating imaging, genetic, and biochemical markers can estimate patient-specific responses to candidate drugs, thereby informing precision dosing and adaptive therapy adjustments [77, 80, 95]. Such biomarker-guided approaches enable therapeutic escalation or de-escalation based on anticipated disease trajectories, addressing the inadequacies of the traditional “one-size-fits-all” model of treatment [76, 79, 91]. This biomarker-guided personalisation directly addresses the heterogeneity of disorders such as AD, PD, and ALS, moving decisively beyond the traditional “one-size-fits-all” approach and by improving diagnostic precision, enhancing prognostic modeling, streamlining therapeutic pipelines, and enabling individualised treatment strategies, AI consistently outperforms traditional empirical methodologies, establishing itself as indispensable to the future of precision neurology [76, 77]. Figure 3 shows the role of AI-based biomarkers and biomarker discovery in neurodegenerative diseases.

### Neuro-oncological diseases

#### Diagnostics: predictive performance, tumour heterogeneity, and early malignancy detection

Conventional MRI, histopathology, and single-feature biomarker pipelines have improved tumour classification but remain insufficient for capturing the biological, spatial, and molecular heterogeneity of gliomas and glioblastomas [96, 97]. Subjective interpretation, sampling bias, and reliance on isolated metrics limit reproducibility, early malignancy detection, and consistent molecular stratification across centres [9, 98].

AI-based diagnostic frameworks add clinical value primarily by addressing these limitations rather than by technical novelty. Across multi-centre studies, DL–based segmentation and radiomic pipelines demonstrate substantially improved reproducibility, with Dice similarity coefficients typically exceeding 0.85–0.90, and marked reductions in inter-observer variability compared with manual contouring [34]. Volumetric assessment reliability is correspondingly enhanced, supporting more consistent longitudinal monitoring and response evaluation [99, 100].

Radiogenomic models capable of inferring key molecular alterations, such as IDH mutation status, MGMT promoter methylation, and 1p/19q codeletion, from routine imaging are particularly impactful when biopsy is high-risk or infeasible. Reported diagnostic performance across independent cohorts typically falls within AUROC ranges of ~ 0.80–0.95, with sensitivities and specificities frequently exceeding 75–85%, indicating clinically meaningful discriminatory power rather than marginal gains [101]. Importantly, these models capture subtle spatial and textural patterns invisible to visual assessment, enabling earlier identification of malignant transformation and biologically aggressive subregions [96, 102].

However, clinical translation remains constrained by heavy reliance on retrospective datasets, scanner- and centre-specific variability, and limited prospective validation. AI does not inherently resolve variability unless data harmonisation and standardised evaluation frameworks are implemented. Initiatives such as AI-RANO represent a critical advance by embedding AI tools into trial-ready, standardised response criteria, improving cross-centre comparability, consistency of survival prediction, and reproducibility of progression assessment [103, 104].

At present, AI-based diagnostic biomarkers should be regarded as precision-enhancing augmentation tools, not replacements for expert judgement. Their long-term clinical value depends on robust prospective validation, interoperability across platforms, and clear demonstration of improved patient-centred outcomes rather than solely high analytical performance metrics [9, 97].

#### Drug discovery and personalising treatment

Drug development in neuro-oncology is marked by high attrition rates, extended timelines, and frequent translational failure, largely driven by profound tumour heterogeneity and suboptimal patient stratification [92, 105]. AI-driven platforms integrating multi-omic, radiomic, and histopathological data fundamentally shift this paradigm by enabling biology-informed target discovery, drug prioritisation, and rational cohort selection at scales unattainable through empirical approaches alone [106–108].

In gliomas,particularly glioblastoma, deep learning applied to radiogenomic and multi-omic datasets has identified novel therapeutic targets, predicted synergistic drug combinations, and refined stratification biomarkers critical for early-phase trial success. Predictive models integrating IDH1/2 mutations, EGFR amplification, and MGMT promoter methylation with radiomic phenotypes achieve AUROC values frequently above 0.80 for treatment-response prediction, substantially outperforming single-modality approaches [105]. This enables tighter alignment between tumour biology and targeted therapeutic strategies, reducing biological noise in clinical trials [106, 107].

For personalised therapy, AI-based prognostic models synthesising imaging, histopathological, and molecular biomarkers generate individualised survival estimates and response probabilities, with reported concordance indices commonly in the 0.70–0.85 range, supporting informed treatment selection over empiric escalation [9]. Incorporation of dynamic biomarkers, including circulating tumour DNA and longitudinal radiomic signatures, further enables adaptive treatment modification, reducing exposure to ineffective therapies while maximising therapeutic benefit [34, 102].

**Fig. 4:**
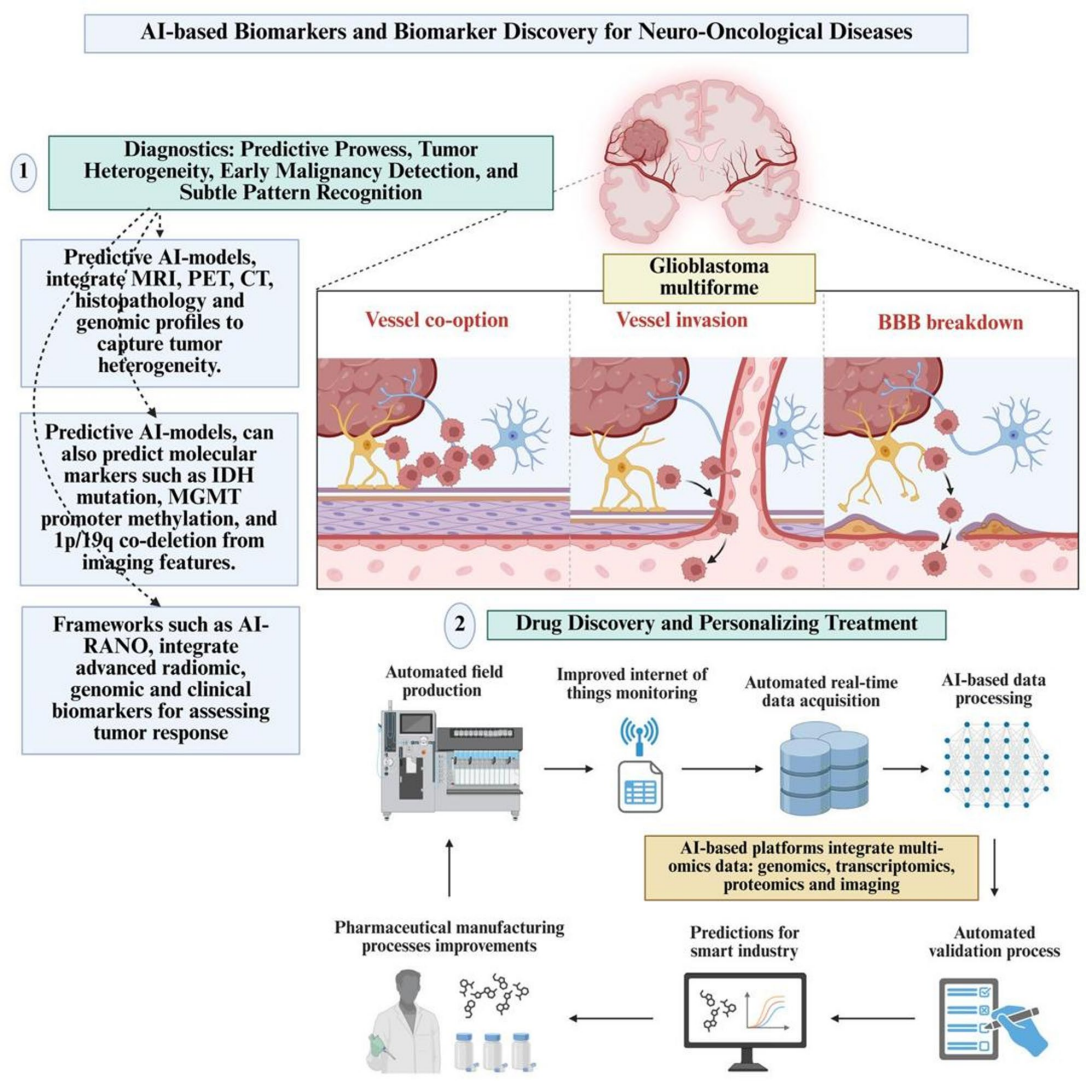
The role of AI-based biomarkers and biomarker discovery in neuro-oncological diseases. AI, artificial intelligence; AI-RANO, artificial intelligence response assessment in neuro-oncology; BBB, blood–brain barrier; CT, computed tomography; IDH, isocitrate dehydrogenase; MGMT, O-6-methylguanine-deoxyribonucleic acid methyltransferase; MRI, magnetic resonance imaging; PET, positron emission tomography. Created with Biorender.com

Standardisation initiatives such as AI-RANO further strengthen this ecosystem by linking AI-derived biomarkers with progression-free and overall survival endpoints, improving trial comparability and predictive consistency across institutions [103, 104]. Collectively, these applications demonstrate that AI-driven biomarker discovery in neuro-oncology directly informs drug development, adaptive trial design, and precision-guided therapy, moving the field beyond descriptive analytics toward clinically actionable decision support [109]. Figure 4 summarises the role of AI-based biomarkers and biomarker discovery in neuro-oncological diseases.

### Epilepsy and seizures

#### Understanding epilepsy etiology, seizure detection and prediction

EEG-based approaches remain central to identifying electrophysiological biomarkers, such as interictal epileptiform discharges (IEDs) and high-frequency oscillations (HFOs). These have been associated with cortical hyperexcitability and seizure generation [110, 111]. IEDs manifest as brief spikes or longer sharp waves, while HFOs indicate hyperexcitable tissue and frequently precede seizures [112]. Incorporating ML and DL techniques into electrophysiological analyses enables a more refined characterisation of pathological features, ranging from microstate alterations to connectivity disturbances that correlate with the localisation of the epileptogenic zone [113–115].

Early work applying neural network classifiers to EEGs demonstrated that AI could reliably recognise epileptiform spike patterns with accuracies up to 94% [116]. Subsequent studies moved beyond spike interpretation towards microstate-level analysis, yielding accuracies of 87% and AUC of 0.94, suggesting strong discriminative ability [117]. Notably, model performance varies substantially across frequency bands, with higher discriminative power in α-bands, β-bands and y-bands, suggesting that epileptogenic processes are preferentially contained in these specific signal components, rather than uniformly across EEG signals [117]. In this way, AI-based analysis could guide more thorough EEG analysis and support the discovery of novel EEG biomarkers.

At a network level, connectivity-based AI models extend these findings by demonstrating that dynamic changes in neural networks, particularly during the transition from pre-ictal to ictal states carry strong predictive value for both seizure onset and surgical outcomes, with some analyses reporting AUC values of 0.9 and classification accuracy of 95% [115]. This not only reinforces the view that epilepsy is a network-based disorder rather than due to focal abnormalities, but also enables mechanistic insight into the networks responsible for generating epileptogenic signals, with direct relevance for surgical targeting and personalised intervention approaches [115].

Automated seizure detection aims to provide early warnings to reduce risk, whilst minimising patient burden from false alarms, making robustness a central requirement. Early AI systems demonstrated that CNNs could reliably detect interictal epileptiform discharges in scalp EEG [118], however failed to demonstrate robustness to uncontrolled EEGs with real-world noise [119]. Subsequent methodological advances shifted towards multiclass classification by integrating EEGs with contextual data such as video recordings and in doing so demonstrated substantially improved discrimination between seizure-related and non-epileptic events in multicentre prospective studies (AUC 0.98, false positives as little as 0.16 per minute). [120, 121] This reduction in false alarms is clinically significant for continuous monitoring applications, where excessive false positives will deter patient adherence and therefore undermine usability.

Building on this, architectural changes in models have been implemented to improve seizure detection through more effective modelling of AI systems. One-dimensional CNNs enable automated extraction of discriminative patterns across brain regions with accuracies of 86–97% [120], whilst ensemble detectors combining multiple algorithms exhibit similar accuracy and improved sensitivity. [122] Attention-based networks resulted in improvements in accuracy from 87% to 89.3%, as well as AUC from 0.86 to 0.88 relative to traditional ML models; whilst this represents a modest statistical improvement, it is clinically relevant given that such improvement equates to fewer missed seizures [123].

Beyond this, several studies have incorporated Benford law derived features to models to detect deviations from the expected structure of EEGs, as a means to distinguish ictal from non-ictal activity. In doing so, they have achieved AUCs of up to 0.95, reflecting near-perfect level of diagnostic separation [124].

Recurrent and graph-based architectures have extended seizure modelling beyond event detection toward subject-independent forecasting. [125] Support vector machines (SVMs) that integrate spikes, HFOs and phase-amplitude coupling have achieved localisation AUC values of 0.73. [126] Similarly, models incorporating amplitude-envelope correlation and phase-locking features predicted seizure onset zones which high accuracy (AUC 0.91), yet patient-level performance remained substantially lower (AUC 0.69), underscoring persistent challenges in cross-subject generalisability. [114] More recent work incorporating hyperbolic space into the geometric analysis of structural brain networks significantly enhanced predictive power for postoperative outcomes in patients with temporal lobe epilepsy, achieving an AUC of 0.9, suggesting that richer geometric representations may partially mitigate these limitations in selected surgical contexts [127].

Collectively, these findings indicate that while AI-based models can achieve high performance under controlled or patient-specific conditions, robust generalisation across individuals remains a widespread barrier to clinical deployment, particularly for prognostic and surgical decision making.

#### Seizure classification

AI-driven seizure classification has improved the differentiation of epilepsy subtypes by reducing reliance on subjective EEG interpretation and enabling consistent pattern recognition across large datasets. When trained on high-density EEG, DL classifiers can reliably distinguish focal from generalised epileptiform activity, addressing a key diagnostic challenge in routine clinical workflows [120, 128].

Beyond seizure phenotype classification, AI models have increasingly been applied to spatial localisation of epileptogenic networks; a task highly relevant to surgical planning. Approaches leveraging high-frequency oscillations from stereo-EEG have demonstrated the ability to localise epileptogenic zones with clinically actionable precision, supporting real-world applicability in pre-surgical evaluation [129].

More recent work has shifted from event-based detection toward network-level representations of brain dynamics, Microstate-based models capture transient, large scale EEG patterns and have shown utility in distinguishing patients with temporal lobe epilepsy from healthy controls, suggesting that seizure classification benefits from modelling distributed network states rather than isolated discharges. [130] Traditional ML approaches, including SVMs trained on microstate features, further support this paradigm by achieving robust classification performance across heterogenous EEG signals [117].

Large, multi-label DL systems extend these advances by jointly classifying seizures, interictal spikes, and artefacts, reducing false alarms compared with binary detectors, improving clinical usability in continuous monitoring settings [131].

Collectively, these approaches illustrate a progression from coarse seizure categorisation toward biologically informed classification frameworks, although their clinical impact remains contingent on data quality, acquisition modality, and prospective validation.

#### Diagnostic sensitivity and specificity performance metrics

The diagnostic performance of epilepsy and seizures requires careful interpretation beyond overall accuracy, as clinically meaningful deployment relies on balancing sensitivity against false-positive burden. Sensitivity quantifies the proportion of seizures that are correctly detected, while specificity reflects how often non-seizure segments are correctly ignored. A critical challenge in deploying AI for epilepsy diagnostics is achieving high sensitivity while minimising false positives. Temporal-convolutional detectors have achieved an F1 score of 0.75, with a false-positive rate of approximately 0.19 per minute, indicating reasonable event detection performance but a non-reassuring alarm burden that may disrupt clinical workflows during patient monitoring [119]. Importantly, meta-analyses reveal that cross-validated models can overestimate real-world performance, particularly for seizure recurrence prediction where AUC values of ~ 0.63–0.67 are reported. [128]. These values suggest only modest predictive ability beyond chance, as a score of 0.5 would be no better than random guesses. These findings underscore the distinction between controlled detection performance and clinically robust prognostic utility. Prospective validation studies further demonstrate that multimodal AI detection pipelines can achieve clinical-grade performance across diverse patient cohorts [121]. However, the heterogeneity of EEG data sources and labelling practices can influence reported accuracy, highlighting the need for standardised benchmarks. Recent studies emphasise not only classical performance measures, but also the clinical interpretability of detection outcomes [110, 111]. Future research must therefore strike a balance between algorithmic precision and clinical usability to ensure widespread adoption in routine care.

#### Evaluating drug efficacy, predicting drug responses and optimising personalised treatment

Accurate prediction of therapeutic response remains a major challenge in the management of epilepsy, as treatment efficacy varies widely across patients and seizure phenotypes. ML models trained on EEG biomarkers have shown promise in stratifying patients according to their likelihood of experiencing seizure recurrence or responding to drugs [132]. Advanced DL models, particularly those incorporating non-linear biomarkers such as EEG features based on Benford's law, offer new ways of predicting pharmacological outcomes by capturing electrophysiological features that may correlate with drug responsiveness [124]. While these approaches remain largely experimental, they demonstrate the potential of AI to move beyond binary detection, and transform epilepsy treatment into a more predictive and personalised approach.

Furthermore, personalised therapy seeks to adapt dynamically to evolving patient networks. AI-driven systems for the automatic detection of pathological oscillations [122] and neuromorphic spiking neural networks have demonstrated the capacity for real-time clinical deployment, thereby enabling adaptive and proactive therapeutic strategies [133]. For example, a neuromorphic spiking neural network that detects ripple-band oscillations has been shown to identify active epilepsy with ~ 80% accuracy [133]. Furthermore, oscillation rates were strongly correlated with seizure frequency (ρ ≈ 0.90, R^2^ ≈ 0.76), indicating a very strong statistical link. Similarly, graph-convolutional approaches based on microstate dynamics have achieved prediction accuracies above 98% with false-positive rates of around 0.021 per hour [125].

Despite these advances, most AI-based models for drug response prediction and adaptive therapy are yet to be externally validated. Robust, prospective validation, integration with clinical decision pathways and regulatory oversight will be essential before such systems can meaningfully inform routine epilepsy care.

### Neurodevelopmental disorders

#### Enhancing diagnostics with AI-based biomarkers

AI-based network models have identified key regulators associated with ASD, such as TP53, TNF and miR-155-5p. This highlights immune and neuronal signalling dysfunction [134]. Blood RNA sequencing approaches have yielded predictive gene signatures, with one 10-gene panel achieving 82% classification accuracy [135]. Similarly, toddler blood messenger ribonucleic acid (mRNA) signatures analysed using LASSO and neural networks produced 21-gene classifiers with an AUC of 0.88 and 86% accuracy [136].

Circulating miRNAs are particularly promising due to their stability in body fluids. A DL framework achieved 95% cross-validation accuracy and 81% independent test accuracy for diagnosing ASD based on dysregulated micro ribonucleic acid (miRNA) biomarkers [137]. At the proteomic level, a combination of mass spectrometry and machine learning identified a 10-protein plasma panel with an AUC of approximately 0.81, reflecting extracellular matrix and immune pathways relevant to ASD [138].

Gut microbiome dysbiosis has also been implicated in ASD. AI models that integrate metagenomic sequencing and SHAP-based feature attribution have identified Prevotella sp. 109 as a leading ASD biomarker. A compact 17-feature microbiome signature achieved 99% classification accuracy using AdaBoost, demonstrating its strong potential for clinical translation; however, larger-scale validation is still required [139].

Neuroimaging and electrophysiology also play a pivotal role. EEG provides robust early biomarkers for NDDs, with systematic reviews showing accuracies of 85–99% in ASD and attention-deficit/hyperactivity disorder (ADHD), and identifying the 9–12 month window as crucial for early ASD biomarker detection [140]. A CNN trained on EEG biomarkers achieved 97.7% accuracy in paediatric ADHD classification [141]. Resting-state fMRI combined with XGBoost revealed abnormal cingulo-opercular and default-mode interactions as ASD biomarkers [142]. Exploratory work on audiovisual integration networks (AVINs) revealed altered connectivity patterns in ADHD, with classifiers achieving 74% diagnostic accuracy [143]. Fusion of resting-state functional magnetic resonance imaging (rs-fMRI) with phenotypic data further improved ASD classification accuracy to 83%, with explainable artificial intelligence (XAI) identifying superior frontal and precuneus biomarkers [144].

Beyond these modalities, an ML analysis of retinal fundus imaging achieved AUROC values of 95–97% for ADHD screening and stratified executive function deficits in visual attention [145]. Case–control and multimodal studies have also highlighted the potential of hybrid diagnostic pipelines. The ASA system, which uses optimised DNNs, achieved an accuracy of approximately 99% for ASD classification [146]. Meanwhile, metabolic biomarker case–control studies have demonstrated the feasibility of clinical implementation [147].

#### AI-based biomarkers for personalised treatment

AI-driven biomarkers improve diagnosis and enable personalised therapy. Salivary metabolomic profiling in children receiving medical cannabis revealed distinct response biomarkers via gradient boosting models, identifying plant metabolites linked to clinical outcomes. These signatures can be used as pharmacodynamic biomarkers to inform adaptive dosing strategies [148].

Electrophysiology and neuroimaging biomarkers facilitate stratification for personalised interventions. For instance, attention auto-encoding networks have been shown to achieve an accuracy rate of 98.9–99.9% in the diagnosis of ADHD, while also highlighting functional connectivity biomarkers that explain the underlying neurobiology [149]. Similarly, EEG and rs-fMRI allow clinicians to monitor treatment response and predict outcomes, with XAI models providing the interpretability necessary for informed decision-making [140, 144].

#### Drug discovery and mechanistic insights

The combination of meta-omics and explainable AI has advanced the discovery of biomarkers into the domain of therapeutic development. Across large ASD cohorts, 342 robust differentially expressed genes were identified, with XAI highlighting genes such as MID2 and the involvement of the fibroblast growth factor receptor pathway as potential therapeutic targets [150]. These biomarkers provide mechanistic insights that can inform the design of targeted therapies and the stratification of clinical trials based on biomarkers.

Proteomic biomarkers identified via mass spectrometry, such as the 10-protein plasma panel associated with the extracellular matrix and immune processes, may also yield insights into the biological pathways underlying ASD [138]. Similarly, microRNA- and mRNA-based signatures can highlight molecular networks linked to specific drug targets and intervention pathways [136, 137].

## Discussion of challenges and prospects of AI-based biomarkers for CNS disorders

### Interpretability and transparency

Although DL models for neuroimaging-based diagnosis of neurological disorders can identify subtle biomarker patterns, their complexity makes them difficult to interpret. One publication describes how XAI techniques such as SHAP, LIME, Grad-CAM and layer-wise relevance propagation can reveal which brain regions and features are driving predictions. This enables clinicians to understand why a scan is classified as AD or the control group [151]. However, another group has noted that, despite recent progress, interpretability, transparency and generalisation remain 'vital issues' for neuroimaging AI. They warn that models trained on data from one hospital often fail when tested on unseen data from other hospitals, and highlight that the 'right to explanation' is emerging in legislation [152]. They argue that decisions affecting patient care should not rely solely on black-box predictions, emphasising that explainability is crucial for legal compliance and trust.

Additional ethical concerns include automation bias causing clinicians to place too much trust in AI output. Meanwhile, legislation such as the 21st Century Cures Act stipulates that clinical AI tools must be explainable to clinicians and patients [153]. Heat-map-based visualisation and structured diagnostic reports have been proposed to help radiologists identify errors and refine models [153]. A systematic review highlights that, while black-box models remain popular, they require post-hoc explainability. When white-box and black-box models perform similarly, interpretable models should be favoured in order to foster clinician trust [154]. Taken together, these findings emphasise that transparent AI is not only a scientific goal, but also a regulatory and ethical requirement.

### Methodology and datasets

AI-based biomarker discovery lies at the interface of neuroimaging, multi-omics and clinical data science; however methodological and dataset constraints continue to hinder progress. ML and DL models frequently overfit when trained on small or homogeneous datasets, performing well within a single institution yet failing to generalise across hospitals, scanners, or patient populations, leading to false discoveries and irreproducible findings [47, 152]. Similar limitations occur with electronic health record (EHR)-based biomarker discovery, where availability varies by age, race and socioeconomic background, while missing laboratory tests, mislabelled records, and non-standardised documentation introduce systematic biases [155, 156]. Without careful harmonisation, these weaknesses significantly undermine the reliability of biomarker research.

Despite these limitations, methodological developments demonstrate clear promise. DL approaches and generative models have identified complex disease-related gene-expression patterns and protein structures, including in ALS, that would otherwise remain undetected [79]. Integrating genomics, transcriptomics, proteomics, metabolomics, imaging, and clinical data enables models to better capture the multifactorial nature of neurodegeneration and to develop biologically meaningful patient stratification systems [157]. However, preventing overfitting demands rigorous cross-validation pipelines, careful feature selection, interpretable model outputs, and explicit confidence reporting to strengthen clinical trust [76]. Furthermore, because adaptive algorithms may drift over time, regulatory evaluation frameworks emphasise assessment of training data characteristics, validation cohorts, algorithmic evolution and performance monitoring [109].

Progress also depends on improving dataset quality. Reliance on single-centre, homogeneous cohorts continues to limit generalisability, particularly for heterogeneous CNS disorders [76]. Large, longitudinal, diverse datasets are essential, driving initiatives across research centres to broaden recruitment and representation [109]. However, standardisation, annotation, and privacy concerns remain labour-intensive barriers to collaboration. Federated learning frameworks provide a realistic solution by allowing distributed model training without sharing raw data [158]. Ultimately, improving methodological rigour, enhancing cohort diversity, and enabling secure multicentre collaboration are crucial for ensuring future AI-derived biomarkers translate meaningfully into clinical practice [76, 109].

### Clinical integration and accuracy

Despite impressive research results, integrating AI-driven biomarker tools into routine clinical workflows remains challenging. Although real-world evaluations of computer-aided detection systems for intracranial haemorrhage report sensitivities above 95% in controlled settings, only a handful of AI tools have been deployed clinically. For instance, one triage system reclassified CT scans from 'routine' to 'stat' and reduced median diagnostic time from 512 to 19 min. However, it prioritised sensitivity over specificity and required radiologist oversight [156]. Automation bias may cause clinicians to rely too heavily on AI output, particularly when they are overwhelmed by their workload, which can lead to them neglecting contradictory human judgement [153]. Concerns about the opacity of many ML systems have prompted governments and regulators to require explainability and data security protocols for clinical AI tools [153].

Integrating smartphone-derived digital biomarkers into clinical trials could reduce sample sizes by around 30% through adaptive trial designs, digital twin modelling and Bayesian updating. However, such approaches require close collaboration between researchers, clinicians, regulators and technology developers [159]. Phenotype-specific algorithms that trigger tailored interventions based on gait variability, typing speed or sleep patterns would also need to be embedded within patient-centred systems. Ultimately, clinical integration will require prospective multi-site validation, regulatory approval and user-centred design to ensure that AI augments, rather than replaces, clinician judgement.

Furthermore, with regard to accuracy, the accuracy reported for AI models in biomarker discovery varies widely and should be interpreted with caution. Studies evaluating ML classifiers trained on CSF biomarkers to differentiate between patients with dementia and healthy controls have found that SVMs and k-nearest neighbour classifiers achieve an accuracy of only 64–69%, with an AUC value of between 0.64 and 0.73. Adding demographic variables does not substantially improve performance [160]. In the context of healthy cognitive ageing, employing blood biomarkers and a gradient-boosting classifier to predict 'SuperAger' status yields a bootstrap accuracy of 75.8% (95% confidence interval 0.66–0.86) and an AUC of 0.739. SHAP analysis reveals that the most influential features are glucose, high density lipoprotein cholesterol, and alanine aminotransferase [161].

For disorders of consciousness following severe brain injury, a combination of functional connectivity and dominant frequency features achieves an accuracy of around 80% (with a sensitivity of 85.7% and a specificity of 71.4%) for traumatic cases, whereas functional connectivity alone achieves an accuracy of around 83.3% (with a sensitivity of 92.3% and a specificity of 60%) for non-traumatic cases [162]. However, even relatively high accuracies may leave substantial room for misclassification, and performance depends strongly on the modality and clinical context. Accuracy alone is insufficient for clinical evaluation: models should be assessed using sensitivity, specificity and AUC metrics, and validated prospectively across cohorts [156]. Improving accuracy will therefore require larger, more diverse datasets, rigorous cross-validation and the integration of multimodal data to capture the heterogeneity of neurological disorders.

### Biases and equity

Biases occur throughout the AI biomarker pipeline. Heavy reliance on datasets such as AD neuroimaging initiative (ADNI) has been criticised due to limited recruitment of non-Alzheimer’s dementias and under-representation of minority populations. Reported accuracy declines when models trained on ADNI-derived datasets are tested on independent cohorts, highlighting a lack of diversity in current biomarker datasets [76]. A prognostic audit of AD progression revealed that logistic regression, SVM and RNN models demonstrated lower sensitivity for Hispanic, Black and Asian participants than for non-Hispanic white participants, although performance by sex was similar. The authors emphasised the need to incorporate fairness metrics such as equal opportunity and equalised odds, and to consider how sampling bias, insufficient sample size, misclassification and algorithm choice contribute to unfairness [163]. In digital biomarker research, many datasets fail to reflect differences across sex, gender, race and cultural background, leading to reduced algorithmic effectiveness across diverse user populations [164].

However, there are meaningful prospects for improvement. The ADNI-4 study now aims to enrol 50–60% of new participants from underrepresented groups, marking a deliberate move toward more representative biomarker datasets [76]. Fairness-aware model training, bias-correction approaches, subgroup performance reporting and fairness-constrained models are increasingly being incorporated into biomarker and diagnostic pipelines for dementia, where explicit optimisation of fairness metrics has been shown to reduce racial and sex-related performance gaps in MRI and EHR based AD prediction [165–167]. Reviews of dementia machine learning datasets further highlight that adherence to FAIR data principles, improved governance and federated multi-cohort collaborations are central to reducing bias and enabling equitable AI-driven biomarker discovery [76, 168]. Moreover, inclusive recruitment strategies and structured equity-auditing frameworks across international AI biomarker consortia provide realistic pathways toward more generalisable and ethically grounded biomarker discovery. Thus, equity remains both an ongoing challenge and a rapidly advancing area of opportunity essential for truly patient-centred precision medicine.

### Economic viability of AI-driven biomarker discovery and precision medicine

The economic rationale for AI-driven biomarker discovery is grounded in its potential to reduce diagnostic burden, optimise clinical trials and enable earlier interventions. A multimodal transformer-based framework integrating demographic, neuropsychological, genetic and imaging data from seven independent cohorts predicted amyloid-beta and tau PET status, allowing individuals unlikely to exhibit pathology to avoid unnecessary imaging. In a cohort of 1,833 participants, this eliminated the need for 587 amyloid PET and 582 tau PET scans, demonstrating substantial potential for healthcare cost savings while preserving diagnostic performance and aiding clinical trial participant selection [169]. Digital biomarker platforms similarly offer opportunities to reduce diagnostic costs through remote monitoring and early detection, potentially making diagnostic access more equitable globally [170]. AI-based tools in ALS and other NDDs are improving clinical trial design efficiency, predicting pharmacokinetics and dosing, and reducing reliance on resource-intensive approaches, aligning with evidence that AI-supported discovery pipelines can be more cost-effective and successful than traditional strategies. Reflecting this, the global AI drug discovery market is projected to grow from US$2.1 billion in 2020 to over US$31 billion by 2030 [79, 171].

However, real-world implementation also faces significant cost challenges. Economic modelling of AI-based MRI triage systems for PD suggests that, although such tools can be cost-effective and reduce long-term diagnostic expenditure, they require substantial up-front investment in infrastructure and careful planning of reimbursement pathways [172]. A systematic review of health economic evaluations of AI imaging tools for real-world neurological diagnostics similarly emphasises the high costs of development, integration and maintenance as major barriers to adoption, and notes that evidence for sustained cost-effectiveness is still limited [173]. Reviews of AI-assisted biomarker discovery in Alzheimer’s disease highlight the invasiveness and cost of many current biomarkers, underscoring the need to prioritise noninvasive, lower cost blood, ocular and digital biomarkers if AI systems are to be economically viable at scale [76, 174, 175]. In parallel, analyses of digital biomarker and wearable neurotechnology platforms for dementia care stress that the costs of building and maintaining secure AI infrastructure, ensuring data privacy and providing equitable access must be incorporated into implementation strategies to avoid exacerbating disparities [176, 177]. Economic viability, therefore, depends not only on potential savings but also on sustainable funding models, reimbursement frameworks and policies that support long-term maintenance of AI biomarker ecosystems.

### Importance of collaborations and interdisciplinary approach

AI-driven biomarker discovery in neurological disorders sits at the intersection of neuroscience, data science, clinical neurology, and bioinformatics. This makes collaboration indispensable. A bibliometric analysis of AI research in neurodegenerative diseases revealed that author collaboration networks exhibit significant intra-group cohesion, yet limited cross-cluster connectivity [178]. The same study also found that clinicians, computer scientists, and bioinformaticians frequently work in disciplinary 'silos' and recommended the establishment of structured interdisciplinary collaboration mechanisms to bridge the gap between clinical expertise and computational innovation [178]. Similarly, a scoping review of digital biomarkers in AD revealed uneven contributions from different disciplines: neurology frequently collaborates with computer and communication engineering, whereas fields such as psychiatry and geriatrics have limited partnerships. Strengthening the ties between medical and engineering disciplines will drive digital biomarker research and optimise future research directions [174].

Consortia can provide such structured collaboration. For example, the European Union-funded MIRIADE project trains early-stage researchers to integrate multi-omics data for dementia biomarkers. During training events, assay developers and bioinformaticians discovered each other’s tools and formed collaborations, integrating data and knowledge to accelerate biomarker development [179]. The authors of MIRIADE highlight how deliberately designed programmes can break down disciplinary barriers and promote data sharing.

On a broader level, Onciul et al. argue that the future of AI in neuroscience hinges on interdisciplinary teams comprising neuroscientists, computer scientists, clinicians, and ethicists [180]. Their review highlights how global data-sharing initiatives and federated learning enable researchers worldwide to contribute datasets while safeguarding privacy, thereby democratising AI tools and ensuring that discoveries reflect diverse populations [180]. The same review also argues that translating AI advances into clinical practice will require ethical vigilance and collaboration across disciplines in order to address concerns such as cognitive privacy and equitable access.

### Roadmap for clinical translation

A clearer framework is required to guide AI-based biomarkers from research environments toward routine clinical use. A practical translational roadmap should include:

- Foundational evidence and validation:
- Biomarker discovery should begin with biologically grounded models supported by rigorous internal validation, transparent reporting, and prospective multi-site external validation to ensure real-world reliability and reproducibility [76, 156].
- Standardisation and harmonisation:
- Consistent imaging protocols, annotation standards, and reporting frameworks are essential, alongside harmonisation strategies and federated learning infrastructures that support secure multi-institutional collaboration without sharing raw patient data [152, 158].
- Regulatory readiness and governance:
- Regulatory frameworks must address adaptive algorithms, accounting for performance drift and ensuring continuous post-deployment monitoring. Evaluation criteria should include training dataset characteristics, algorithm transparency, safety mechanisms, and compliance with explainability legislation [109, 153].
- Clinical workflow integration:
- AI biomarkers should be implemented in clinician-centred systems featuring interpretable outputs, confidence estimates, meaningful visualisation tools and safeguards against automation bias. Co-design with clinicians and patients is essential to promote usability, trust and acceptance [153, 154].
- Equity assurance:
- Deployment must systematically incorporate diverse cohorts, fairness auditing, subgroup performance evaluation and bias mitigation to ensure equitable benefit across populations [76, 163].
- Sustainable real-world implementation:
- Long-term translation requires economic sustainability, data interoperability, cybersecurity, robust infrastructure support and iterative refinement based on real-world outcomes to ensure clinical effectiveness.

## Conclusion

In conclusion, these studies consistently demonstrate that AI can enhance the detection of various neurological diseases and provide meaningful risk stratification by integrating more complex metrics. Based on this evidence, it appears that AI could play a significant role in reducing the incidence of neurological complications and improving personalised treatment. Notably, AI models offer a more accurate means of defining the stability of neurological cases, adding layers of precision to diagnostics that directly inform management decisions. While their generalisability across populations and imaging platforms is yet to be consolidated, future research will require multicentre, prospective trials and the integration of multimodal biomarkers. The development of explainable AI systems will also be necessary to enhance transparency. If these hurdles can be overcome, AI has the potential to evolve from an adjunctive tool to a central tenet of the diagnosis and treatment of neurological diseases in the future.

## Supplementary Information

Supplementary Material 1

## Data Availability

No datasets were generated or analysed during the current study.

## Abbreviations

AI: Artificial intelligence
ML: Machine learning
DL: Deep learning
CNS: Central nervous system
NDDs: Neurodegenerative disorders
AAN: Artificial neural network
MRI: Magnetic resonance imaging
CT: Computed tomography
WHO: World Health Organization
NfL: Neurofilament light chain
ALS: Amyotrophic lateral sclerosis
TBI: Traumatic brain injury
PD: Parkinson's disease
DNA: Deoxyribonucleic acid
RNA: Ribonucleic acid
DISFC: Dual integration stepwise fusion classifier
COA: Clinical outcome assessment
AD: Alzheimer’s disease
ADNI: Alzheimer’s disease neuroimaging initiative
EEG: Electroencephalography
EMG: Electromyography
ASM: Anti-seizure medication
SPaRCNet: Seizure prediction and reliable control network
SCORE: Standardised computer-based organised reporting of EEG
NCCT: Non-contrast computed tomography
AIS: Acute ischemic stroke
NWU: Net water uptake
SBI: Silent brain infarction
SII: Systemic immune inflammatory index
PLR: Platelet-to-lymphocyte ratio
CVT: Central venous thrombosis
CNN: Convolutional neural network
MCI: Mild cognitive impairment
CSF: Cerebrospinal fluid
AUC: Area under the curve
ADHD: Attention-deficit/hyperactivity disorder
ASD: Autism spectrum disorder
MMP-9: Matrix metalloproteinase-9
IEDs: Interical epileptic discharge
HFO: High-frequency oscillation
XAI: Explainable artificial intelligence
AI-RANO: Artificial intelligence response assessment in neuro-oncology
MGMT: O-6-methylguanine-deoxyribonucleic acid methyltransferase
GFAP: Glial fibrillary acidic protein
NSE: Neuron-specific enolase
AUROC: Area under the receiver operating curve
RNA: Ribonucleic acid
EGRR: Epidermal growth factor receptor
PET: Positron emission tomography
IDH: Isocitrate dehydrogenase
SVM: Support vector machine
rs-fMRI: Resting-state functional magnetic resonance imaging
EHR: Electronic health record
AVIN: Audiovisual integration network
miRNA: Micro ribonucleic acid
mRNA: Messenger ribonucleic acid
ASPECTS: Alberta stroke program early CT score
MSMT-DL: Multisequence multitask deep learning
