# Supplementary material for: Artificial intelligence-based biomarkers for the diagnosis and treatment of neurological conditions: a narrative review
Source: Mol Brain. 2026 Mar 7;19:26. doi: 10.1186/s13041-026-01287-1 (PMC13081627; doi:10.1186/s13041-026-01287-1)
Supplement: Supplementary file 1 — Supplementary Material 1 [file 13041_2026_1287_MOESM1_ESM.docx]

Supplementary table 1. Search strings for methodology

| **Databases** | **Search Strings** |
| --- | --- |
| PubMed/ Medline | (("Artificial Intelligence"[MeSH] OR "Machine Learning"[MeSH] OR "Deep Learning"[MeSH] OR "Neural Networks, Computer"[MeSH] OR "Support Vector Machine"[MeSH] OR "artificial intelligence"[tiab] OR "machine learning"[tiab] OR "deep learning"[tiab] OR "neural network*"[tiab] OR "CNN"[tiab] OR "computer vision"[tiab] OR "transfer learning"[tiab] OR "explainable AI"[tiab] OR "XAI"[tiab] OR "radiomics"[tiab] OR "radiogenomics"[tiab]) AND ("Biomarkers"[MeSH] OR "Biological Markers"[MeSH] OR "Precision Medicine"[MeSH] OR "biomarker*"[tiab] OR "biological marker*"[tiab] OR "signature*"[tiab] OR "multi-omics"[tiab] OR "metabolomics"[tiab] OR "proteomics"[tiab] OR "genomics"[tiab] OR "transcriptomics"[tiab] OR "neuroimaging biomarker*"[tiab] OR "digital biomarker*"[tiab] OR "electrophysiological biomarker*"[tiab] OR "biomarker discovery"[tiab]) AND ("Nervous System Diseases"[MeSH] OR "Central Nervous System Diseases"[MeSH] OR "Neurodegenerative Diseases"[MeSH] OR "Stroke"[MeSH] OR "Brain Neoplasms"[MeSH] OR "Epilepsy"[MeSH] OR "Neurodevelopmental Disorders"[MeSH] OR "neurological disorder*"[tiab] OR "CNS disorder*"[tiab] OR "neurovascular"[tiab] OR "stroke"[tiab] OR "intracranial aneurysm*"[tiab] OR "cerebral venous thrombosis"[tiab] OR "CVT"[tiab] OR "neurodegenerative"[tiab] OR "Alzheimer*"[tiab] OR "Parkinson*"[tiab] OR "amyotrophic lateral sclerosis"[tiab] OR "ALS"[tiab] OR "neuro-oncology"[tiab] OR "glioma*"[tiab] OR "glioblastoma*"[tiab] OR "epilepsy"[tiab] OR "seizure*"[tiab] OR "neurodevelopmental"[tiab] OR "autism"[tiab] OR "ASD"[tiab] OR "ADHD"[tiab])) |
| Scopus | (TITLE-ABS-KEY ( "Artificial Intelligence" OR "Machine Learning" OR "Deep Learning" OR "Neural Networks" OR "Support Vector Machine" OR "artificial intelligence" OR "machine learning" OR "deep learning" OR "neural network*" OR "CNN" OR "computer vision" OR "transfer learning" OR "explainable AI" OR "XAI" OR "radiomics" OR "radiogenomics" ) )  AND (TITLE-ABS-KEY ( "Biomarkers" OR "Biological Markers" OR "Precision Medicine" OR "biomarker*" OR "biological marker*" OR "signature*" OR "multi-omics" OR "metabolomics" OR "proteomics" OR "genomics" OR "transcriptomics" OR "neuroimaging biomarker*" OR "digital biomarker*" OR "electrophysiological biomarker*" OR "biomarker discovery" ))  AND (TITLE-ABS-KEY ( "Nervous System Diseases" OR "Central Nervous System Diseases" OR "Neurodegenerative Diseases" OR "Stroke" OR "Brain Neoplasms" OR "Epilepsy" OR "Neurodevelopmental Disorders" OR "neurological disorder*" OR "CNS disorder*" OR "neurovascular" OR "stroke" OR "intracranial aneurysm*" OR "cerebral venous thrombosis" OR "CVT" OR "neurodegenerative" OR "Alzheimer*" OR "Parkinson*" OR "amyotrophic lateral sclerosis" OR "ALS" OR "neuro-oncology" OR "glioma*" OR "glioblastoma*" OR "epilepsy" OR "seizure*" OR "neurodevelopmental" OR "autism" OR "ASD" OR "ADHD")) |
| Embase | ('artificial intelligence' OR 'machine learning' OR 'deep learning' OR 'artificial neural network' OR 'support vector machine' OR 'artificial intelligence':ti,ab,kw OR 'machine learning':ti,ab,kw OR 'deep learning':ti,ab,kw OR 'neural network*':ti,ab,kw OR 'cnn':ti,ab,kw OR 'computer vision':ti,ab,kw OR 'transfer learning':ti,ab,kw OR 'explainable ai':ti,ab,kw OR 'xai':ti,ab,kw OR 'radiomics':ti,ab,kw OR 'radiogenomics':ti,ab,kw) AND ('biological marker' OR 'precision medicine' OR 'biomarker*':ti,ab,kw OR 'biological marker*':ti,ab,kw OR 'signature*':ti,ab,kw OR 'multiomics':ti,ab,kw OR 'metabolomics':ti,ab,kw OR 'proteomics':ti,ab,kw OR 'genomics':ti,ab,kw OR 'transcriptomics':ti,ab,kw OR 'neuroimaging biomarker*':ti,ab,kw OR 'digital biomarker*':ti,ab,kw OR 'electrophysiological biomarker*':ti,ab,kw OR 'biomarker discovery':ti,ab,kw) AND ('neurological disease' OR 'central nervous system disease' OR 'neurodegenerative disease' OR 'cerebrovascular accident' OR 'brain tumor'OR 'epilepsy' OR 'neurodevelopmental disorder' OR 'neurological disorder*':ti,ab,kw OR 'cns disorder*':ti,ab,kw OR 'neurovascular':ti,ab,kw OR 'stroke':ti,ab,kw OR 'intracranial aneurysm*':ti,ab,kw OR 'cerebral venous thrombosis':ti,ab,kw OR 'cvt':ti,ab,kw OR 'neurodegenerative':ti,ab,kw OR 'alzheimer*':ti,ab,kw OR 'parkinson*':ti,ab,kw OR 'amyotrophic lateral sclerosis':ti,ab,kw OR 'als':ti,ab,kw OR 'neurooncology':ti,ab,kw OR 'glioma*':ti,ab,kw OR 'glioblastoma*':ti,ab,kw OR 'epilepsy':ti,ab,kw OR 'seizure*':ti,ab,kw OR 'neurodevelopmental':ti,ab,kw OR 'autism':ti,ab,kw OR 'asd':ti,ab,kw OR 'adhd':ti,ab,kw) |
| CINAHL Plus | ((MH "Artificial Intelligence+") OR (MH "Machine Learning+") OR (MH "Neural Networks, Computer+") OR (MH "Support Vector Machines") OR TI ("artificial intelligence" OR "machine learning" OR "deep learning" OR "neural network*" OR "CNN" OR "computer vision" OR "transfer learning" OR "explainable AI" OR "XAI" OR "radiomics" OR "radiogenomics") OR AB ("artificial intelligence" OR "machine learning" OR "deep learning" OR "neural network*" OR "CNN" OR "computer vision" OR "transfer learning" OR "explainable AI" OR "XAI" OR "radiomics" OR "radiogenomics")) AND ((MH "Biomarkers+") OR (MH "Precision Medicine") OR TI ("biomarker*" OR "biological marker*" OR "signature*" OR "multi-omics" OR "metabolomics" OR "proteomics" OR "genomics" OR "transcriptomics" OR "neuroimaging biomarker*" OR "digital biomarker*" OR "electrophysiological biomarker*" OR "biomarker discovery") OR AB ("biomarker*" OR "biological marker*" OR "signature*" OR "multi-omics" OR "metabolomics" OR "proteomics" OR "genomics" OR "transcriptomics" OR "neuroimaging biomarker*" OR "digital biomarker*" OR "electrophysiological biomarker*" OR "biomarker discovery"))  AND ((MH "Nervous System Diseases+") OR (MH "Neurodegenerative Diseases+") OR (MH "Stroke+") OR (MH "Brain Neoplasms+") OR (MH "Epilepsy+") OR (MH "Developmental Disabilities+") OR TI ("neurological disorder*" OR "CNS disorder*" OR "neurovascular" OR "stroke" OR "intracranial aneurysm*" OR "cerebral venous thrombosis" OR "CVT" OR "neurodegenerative" OR "Alzheimer*" OR "Parkinson*" OR "amyotrophic lateral sclerosis" OR "ALS" OR "neuro-oncology" OR "glioma*" OR "glioblastoma*" OR "epilepsy" OR "seizure*" OR "neurodevelopmental" OR "autism" OR "ASD" OR "ADHD") OR AB ("neurological disorder*" OR "CNS disorder*" OR "neurovascular" OR "stroke" OR "intracranial aneurysm*" OR "cerebral venous thrombosis" OR "CVT" OR "neurodegenerative" OR "Alzheimer*" OR "Parkinson*" OR "amyotrophic lateral sclerosis" OR "ALS" OR "neuro-oncology" OR "glioma*" OR "glioblastoma*" OR "epilepsy" OR "seizure*" OR "neurodevelopmental" OR "autism" OR "ASD" OR "ADHD")) |
| IEEE Xplorer | (("All Metadata":"Artificial Intelligence" OR "All Metadata":"Machine Learning" OR "All Metadata":"Deep Learning" OR "All Metadata":"Neural Network*" OR "All Metadata":"CNN" OR "All Metadata":"Computer Vision" OR "All Metadata":"Explainable AI") AND ("All Metadata":"Biomarker*" OR "All Metadata":"Multi-omics" OR "All Metadata":"Radiomics" OR "All Metadata":"Precision Medicine") AND ("All Metadata":"Neurological" OR "All Metadata":"Stroke" OR "All Metadata":"Neurodegenerative" OR "All Metadata":"Brain Neoplasm*" OR "All Metadata":"Epilepsy" OR "All Metadata":"Neurodevelopmental")) |
| Cochrane Library | (("Artificial Intelligence"[MeSH] OR "Machine Learning"[MeSH] OR "Deep Learning"[MeSH] OR "Neural Networks, Computer"[MeSH] OR "Support Vector Machine"[MeSH] OR "artificial intelligence"[tiab] OR "machine learning"[tiab] OR "deep learning"[tiab] OR "neural network*"[tiab] OR "CNN"[tiab] OR "computer vision"[tiab] OR "transfer learning"[tiab] OR "explainable AI"[tiab] OR "XAI"[tiab] OR "radiomics"[tiab] OR "radiogenomics"[tiab]) AND ("Biomarkers"[MeSH] OR "Biological Markers"[MeSH] OR "Precision Medicine"[MeSH] OR "biomarker*"[tiab] OR "biological marker*"[tiab] OR "signature*"[tiab] OR "multi-omics"[tiab] OR "metabolomics"[tiab] OR "proteomics"[tiab] OR "genomics"[tiab] OR "transcriptomics"[tiab] OR "neuroimaging biomarker*"[tiab] OR "digital biomarker*"[tiab] OR "electrophysiological biomarker*"[tiab] OR "biomarker discovery"[tiab]) AND ("Nervous System Diseases"[MeSH] OR "Central Nervous System Diseases"[MeSH] OR "Neurodegenerative Diseases"[MeSH] OR "Stroke"[MeSH] OR "Brain Neoplasms"[MeSH] OR "Epilepsy"[MeSH] OR "Neurodevelopmental Disorders"[MeSH] OR "neurological disorder*"[tiab] OR "CNS disorder*"[tiab] OR "neurovascular"[tiab] OR "stroke"[tiab] OR "intracranial aneurysm*"[tiab] OR "cerebral venous thrombosis"[tiab] OR "CVT"[tiab] OR "neurodegenerative"[tiab] OR "Alzheimer*"[tiab] OR "Parkinson*"[tiab] OR "amyotrophic lateral sclerosis"[tiab] OR "ALS"[tiab] OR "neuro-oncology"[tiab] OR "glioma*"[tiab] OR "glioblastoma*"[tiab] OR "epilepsy"[tiab] OR "seizure*"[tiab] OR "neurodevelopmental"[tiab] OR "autism"[tiab] OR "ASD"[tiab] OR "ADHD"[tiab])) |
